# Supplementary material for: A Humanized Yeast Phenomic Model of Deoxycytidine Kinase to Predict Genetic Buffering of Nucleoside Analog Cytotoxicity
Source: Genes (Basel). 2019 Sep 30;10(10):770. doi: 10.3390/genes10100770 (PMC6826991; doi:10.3390/genes10100770)

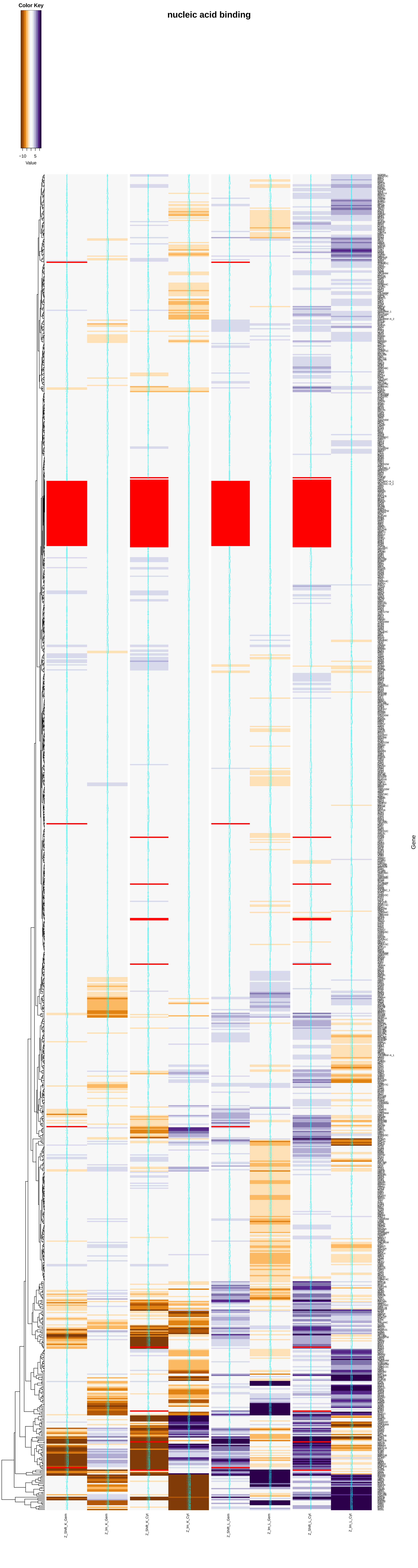
Color Key
−10
5
Value
nucleic acid binding
Z_Shift_K_Gem
Z_Im_K_Gem
Z_Shift_K_Cyt
Z_Im_K_Cyt
Z_Shift_L_Gem
Z_Im_L_Gem
Z_Shift_L_Cyt
Z_Im_L_Cyt
Gene

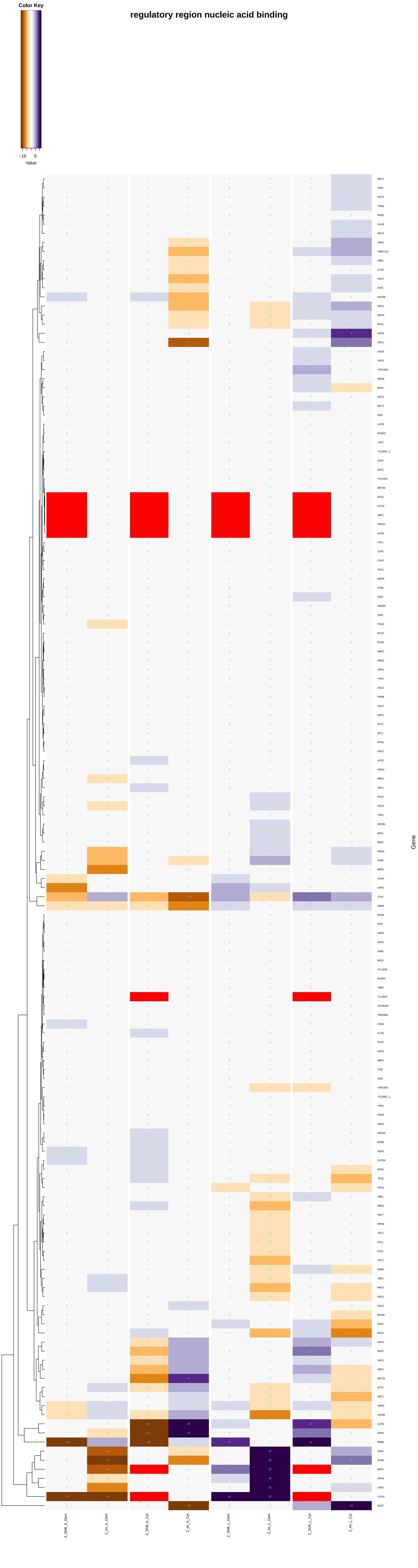
regulatory region nucleic acid binding
Color Key
−10
5
Value
Gene

DNA binding

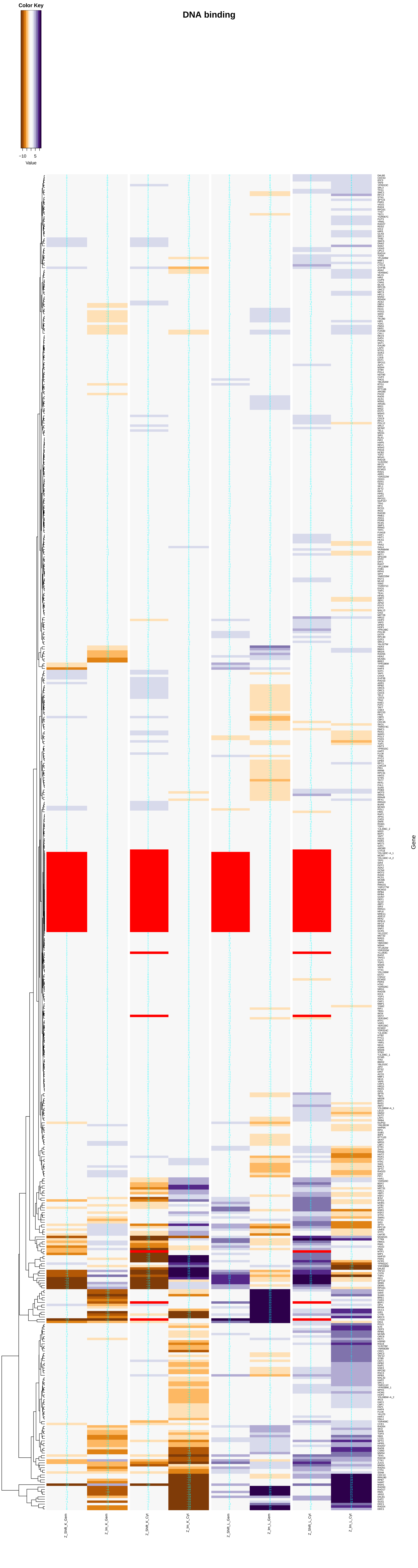
Color Key
−10
5
Value
Gene
Z_Shift_K_Gem
Z_Im_K_Gem
Z_Shift_K_Cyt
Z_Im_K_Cyt
Z_Shift_L_Gem
Z_Im_L_Gem
Z_Shift_L_Cyt
Z_Im_L_Cyt

RNA binding

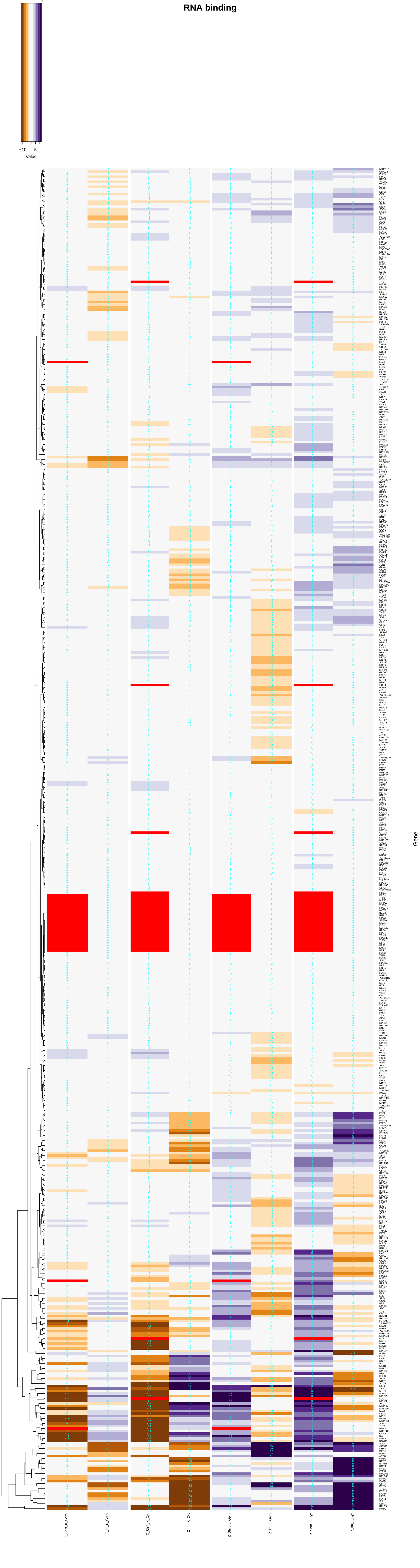

Color Key
−10
5
Value
Gene
Z_Shift_K_Gem
Z_Im_K_Gem
Z_Shift_K_Cyt
Z_Im_K_Cyt
Z_Shift_L_Gem
Z_Im_L_Gem
Z_Shift_L_Cyt
Z_Im_L_Cyt

translation regulator activity, nucleic acid binding

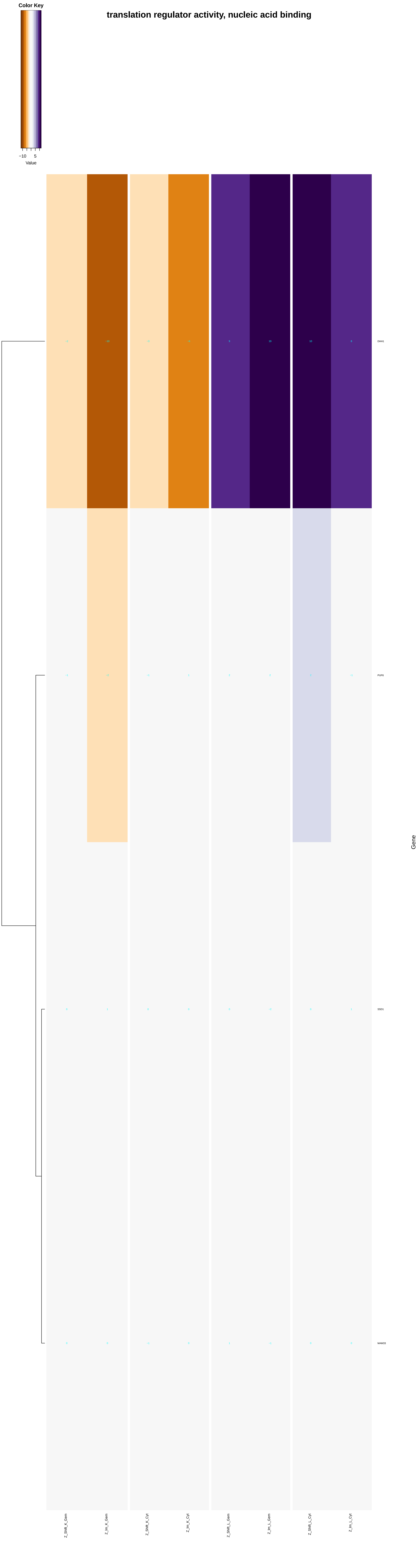

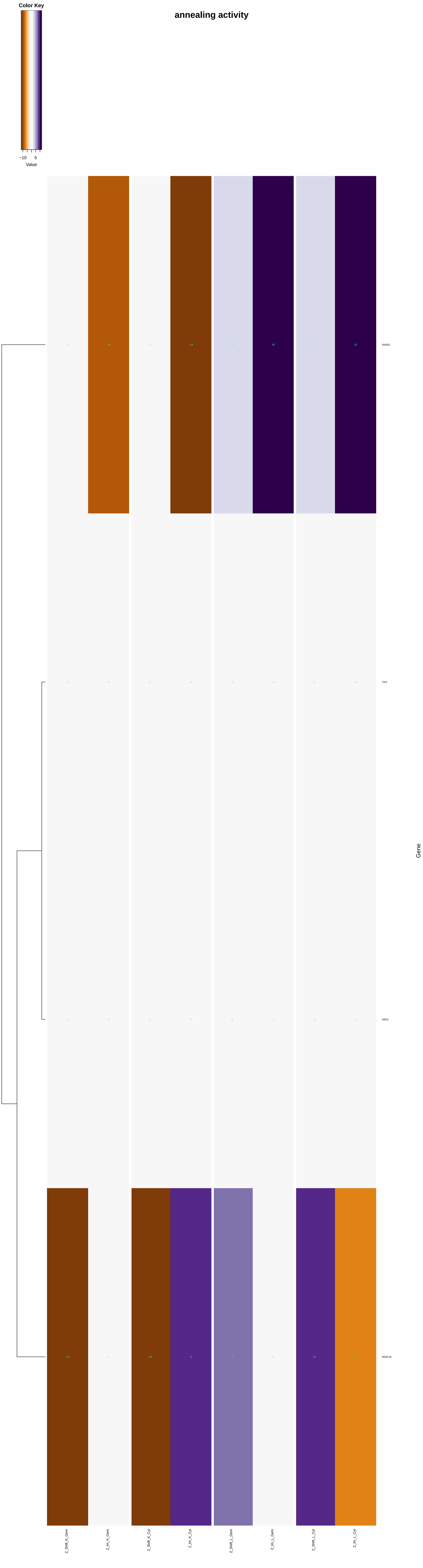

annealing activity
Color Key
−10
5
Value
RAD52
TIF3
DED1
MSS116
Gene
Z_Shift_K_Gem
Z_Im_K_Gem
Z_Shift_K_Cyt
Z_Im_K_Cyt
Z_Shift_L_Gem
Z_Im_L_Gem
Z_Shift_L_Cyt
Z_Im_L_Cyt

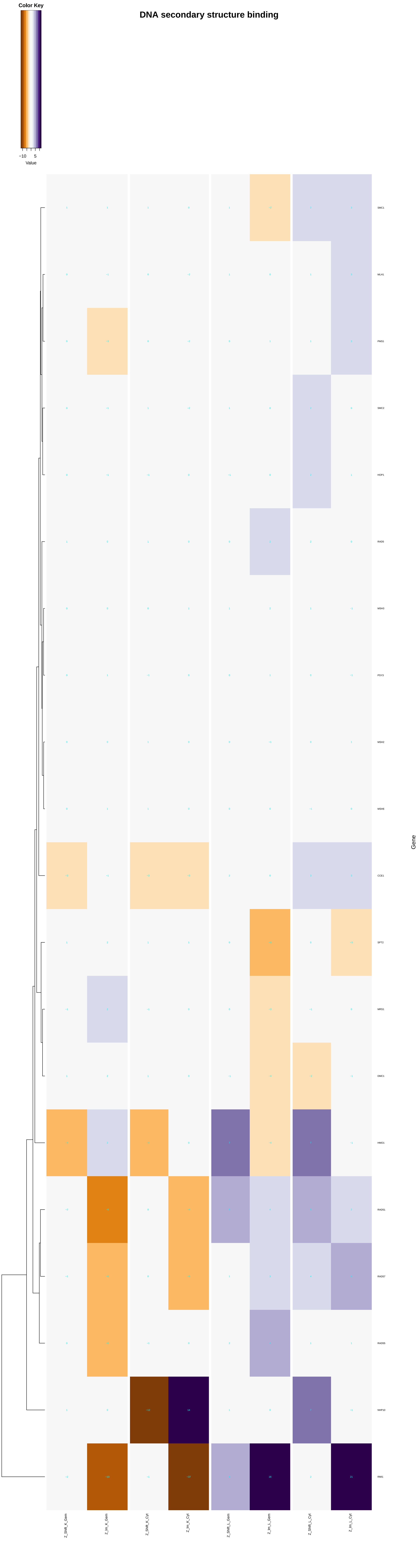
DNA secondary structure binding
Color Key
−10
5
Value
Gene
SMC1
MLH1
PMS1
SMC2
HOP1
RAD5
MSH3
PSY3
MSH2
MSH6
CCE1
SPT2
MRS1
DMC1
HMO1
RAD51
RAD57
RAD55
NHP10
RIM1
Z_Shift_K_Gam
Z_Int_K_Gam
Z_Shift_K_Cyt
Z_Int_K_Cyt
Z_Shift_L_Gam
Z_Int_L_Gam
Z_Shift_L_Cyt
Z_Int_L_Cyt

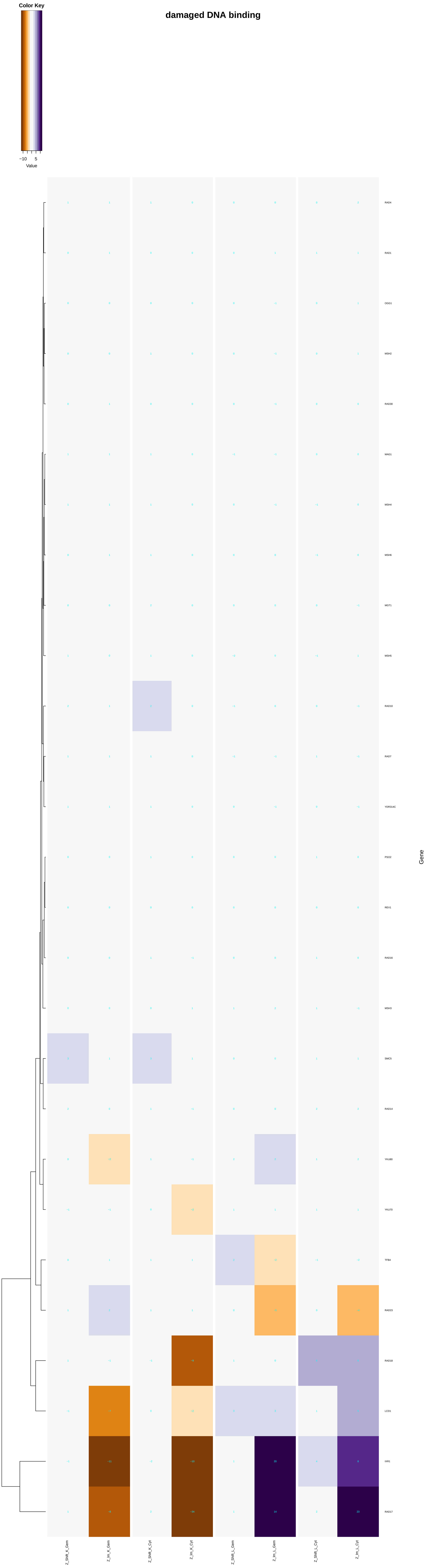

Color Key
−10
5
Value
damaged DNA binding
RAD4
RAD1
OGG1
MSH2
RAD30
MAG1
MSH4
MSH6
MGT1
MSH5
RAD10
RAD7
YOR314C
PSO2
REV1
RAD16
MSH3
SMC6
RAD14
YKU80
YKU70
TFB4
RAD23
RAD18
LCD1
IXR1
RAD17
Gene
Z_Shift_K_Gem
Z_Int_K_Gem
Z_Shift_K_Cyt
Z_Int_K_Cyt
Z_Shift_L_Gem
Z_Int_L_Gem
Z_Shift_L_Cyt
Z_Int_L_Cyt

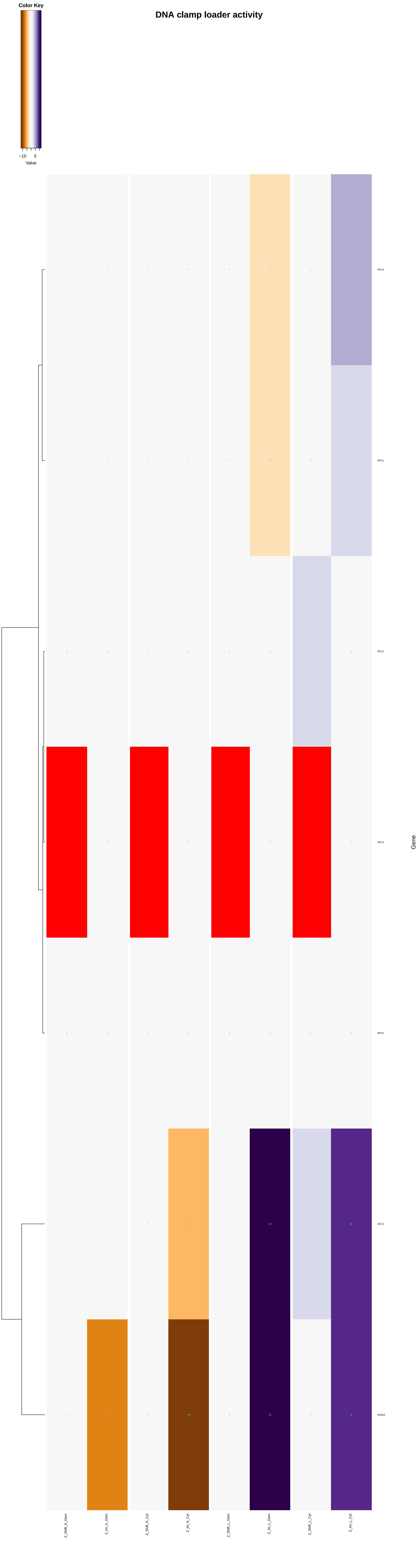
Color Key
−10
5
Value
DNA clamp loader activity
RFC3
RFC1
RFC2
RFC4
RFC5
DCC1
RAD24
Gene
Z_Shift_K_Gem
Z_Int_K_Gem
Z_Shift_K_Cyt
Z_Int_K_Cyt
Z_Shift_L_Gem
Z_Int_L_Gem
Z_Shift_L_Cyt
Z_Int_L_Cyt

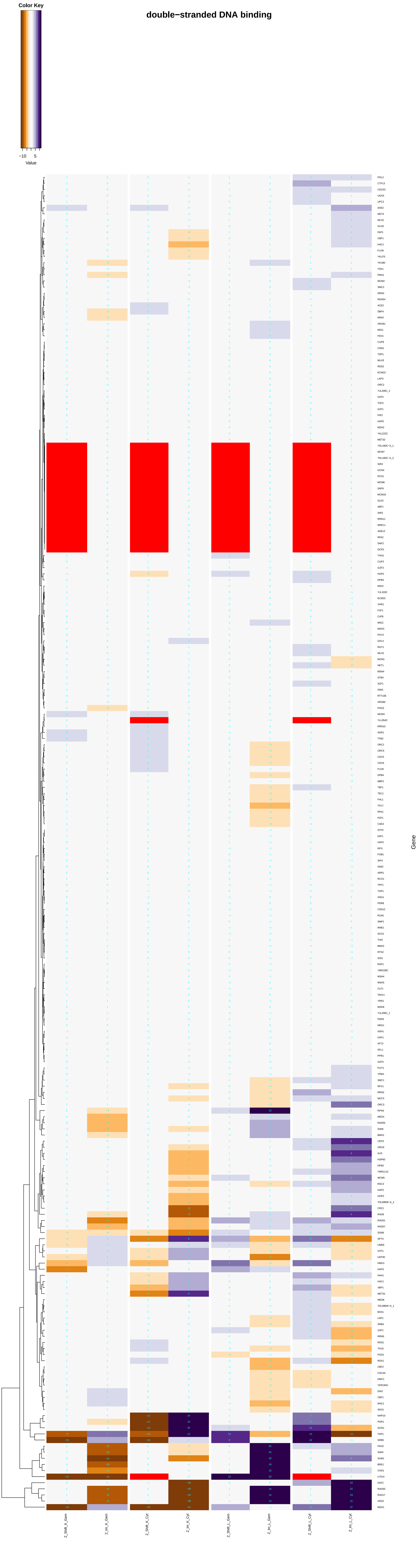
double−stranded DNA binding
Color Key
−10
5
Value
Gene
Z_Shift_K_Gem
Z_Im_K_Gem
Z_Shift_K_Cyt
Z_Im_K_Cyt
Z_Shift_L_Gem
Z_Im_L_Gem
Z_Shift_L_Cyt
Z_Im_L_Cyt

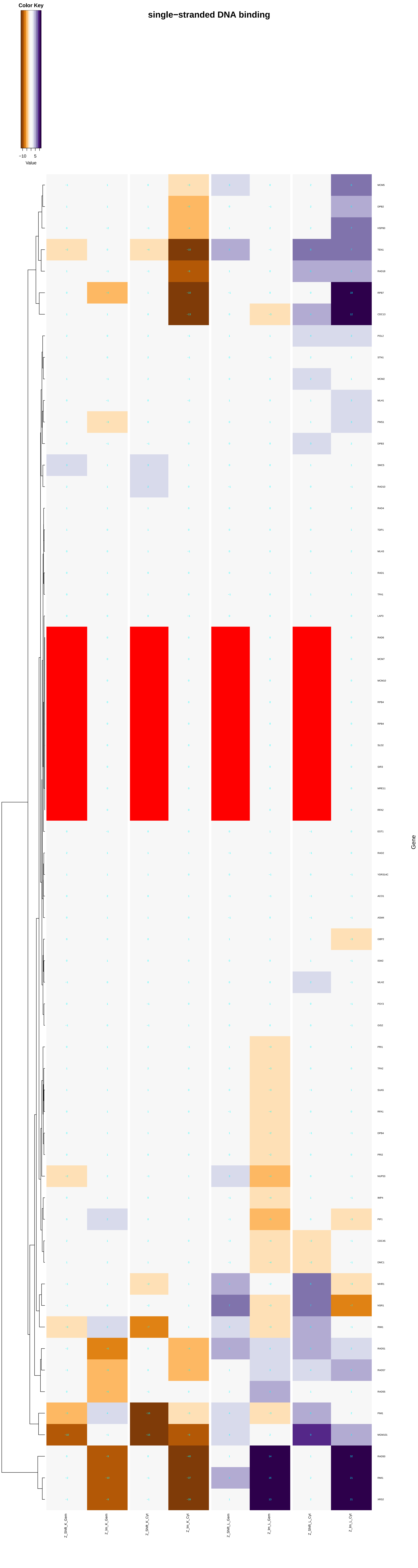

single−stranded DNA binding
Color Key
Value
Gene

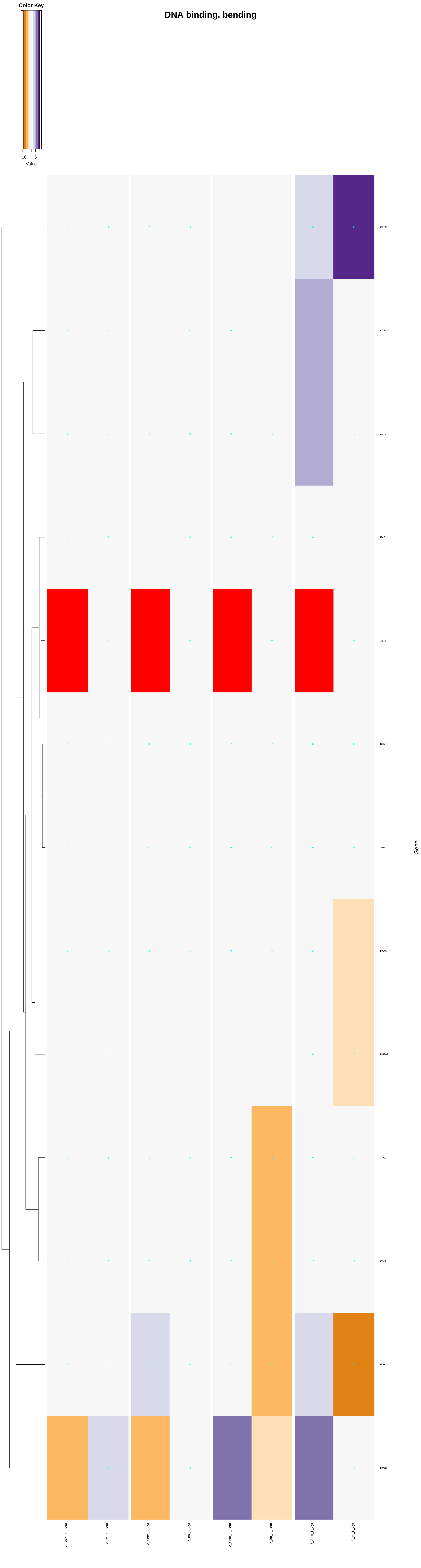
DNA binding, bending
Color Key
−10
5
Value
CEF3
CTF13
ABF2
RAP1
ABF1
RLM1
SMP1
MCM1
NHP6A
TFC7
CBF2
ROX1
HMO1
Gene
Z_Shift_K_Gem
Z_Int_K_Gem
Z_Shift_K_Cyt
Z_Int_K_Cyt
Z_Shift_L_Gem
Z_Int_L_Gem
Z_Shift_L_Cyt
Z_Int_L_Cyt

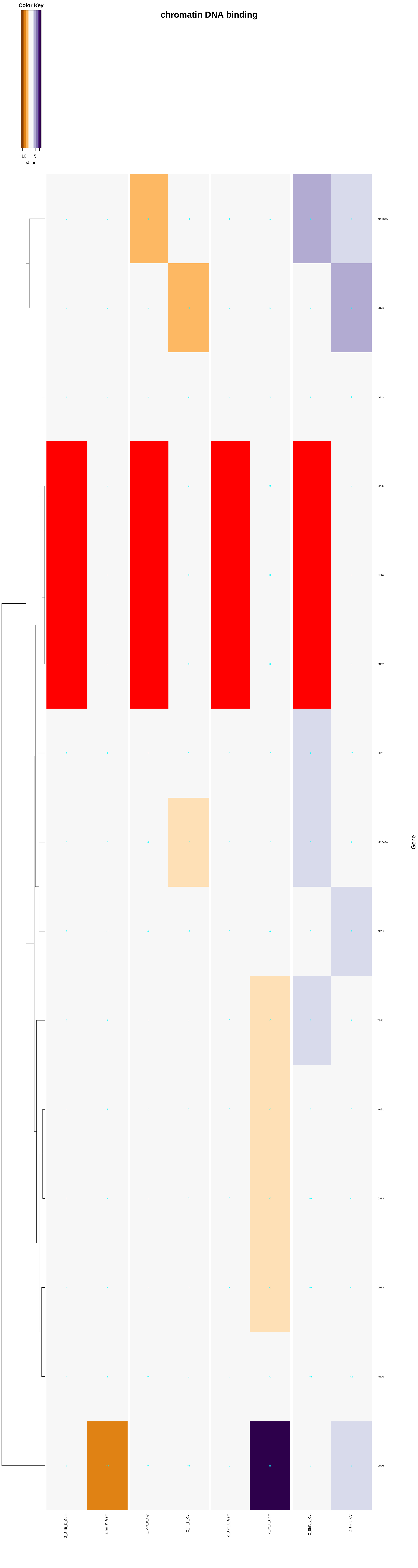
Color Key
−10
5
Value
chromatin DNA binding
YOR456C
SRC3
RAP1
NPL6
GON7
SNF2
HHT1
YFL049W
SRC1
TBF1
KAE1
CSE4
DPB4
RED1
CHD1
Gene
Z_Shift_K_Gem
Z_Int_K_Gem
Z_Shift_K_Cyt
Z_Int_K_Cyt
Z_Shift_L_Gem
Z_Int_L_Gem
Z_Shift_L_Cyt
Z_Int_L_Cyt

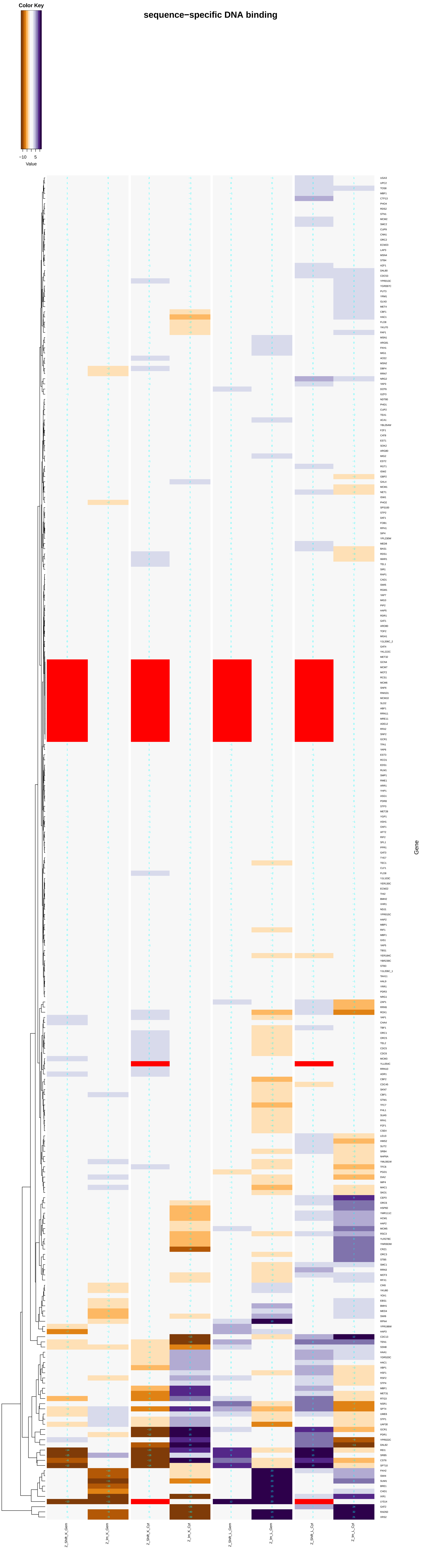

sequence−specific DNA binding
Color Key
−10
5
Value
Gene
Z_Shift_K_Gen
Z_Im_K_Gen
Z_Shift_K_Cyt
Z_Im_K_Cyt
Z_Shift_L_Gen
Z_Im_L_Gen
Z_Shift_L_Cyt
Z_Im_L_Cyt

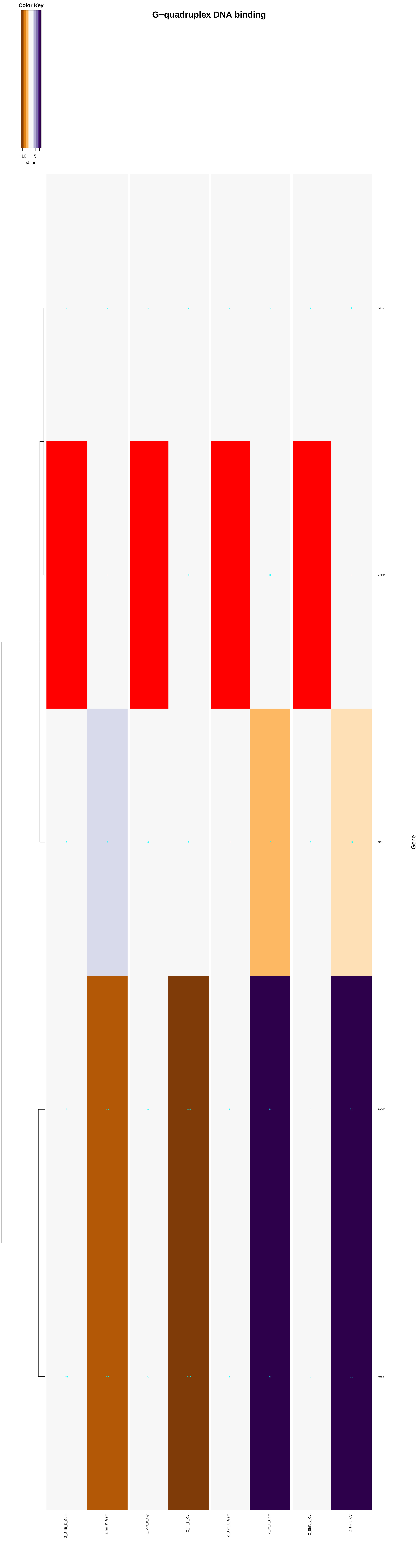
G−quadruplex DNA binding
Color Key
−10
5
Value
RAP1
MRE11
PIF1
RAD50
XRS2
Gene
Z_Shift_K_Gem
Z_Im_K_Gem
Z_Shift_K_Cyt
Z_Im_K_Cyt
Z_Shift_L_Gem
Z_Im_L_Gem
Z_Shift_L_Cyt
Z_Im_L_Cyt

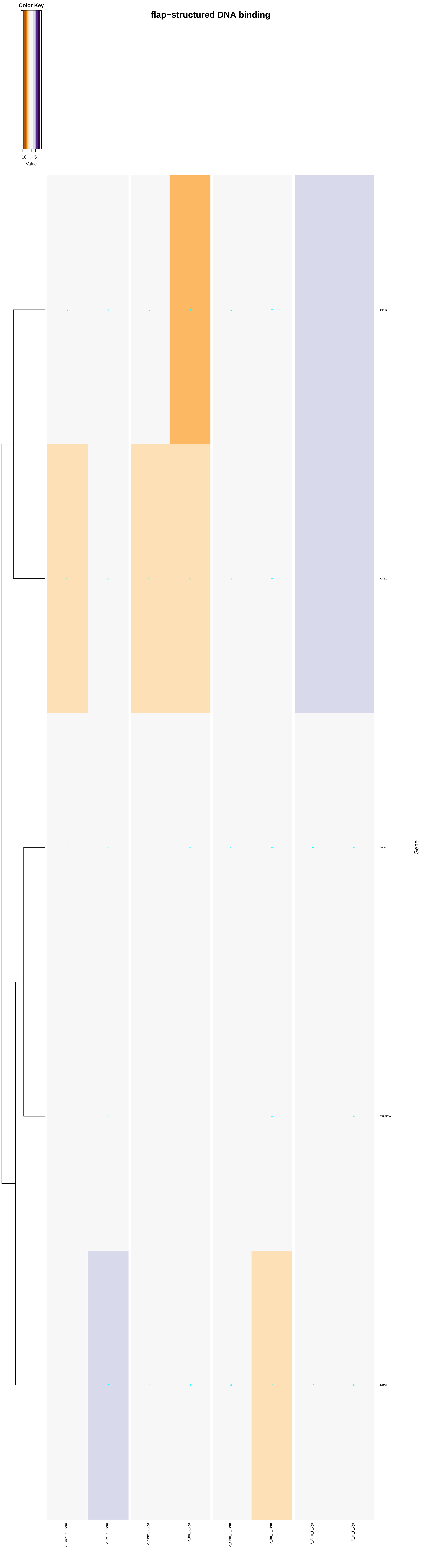

flap−structured DNA binding
Color Key
−10
5
Value
MPH1
CCE1
VTS1
YAL027W
MRS1
Gene
Z_Shift_K_Gem
Z_Im_K_Gem
Z_Shift_K_Cyt
Z_Im_K_Cyt
Z_Shift_L_Gem
Z_Im_L_Gem
Z_Shift_L_Cyt
Z_Im_L_Cyt

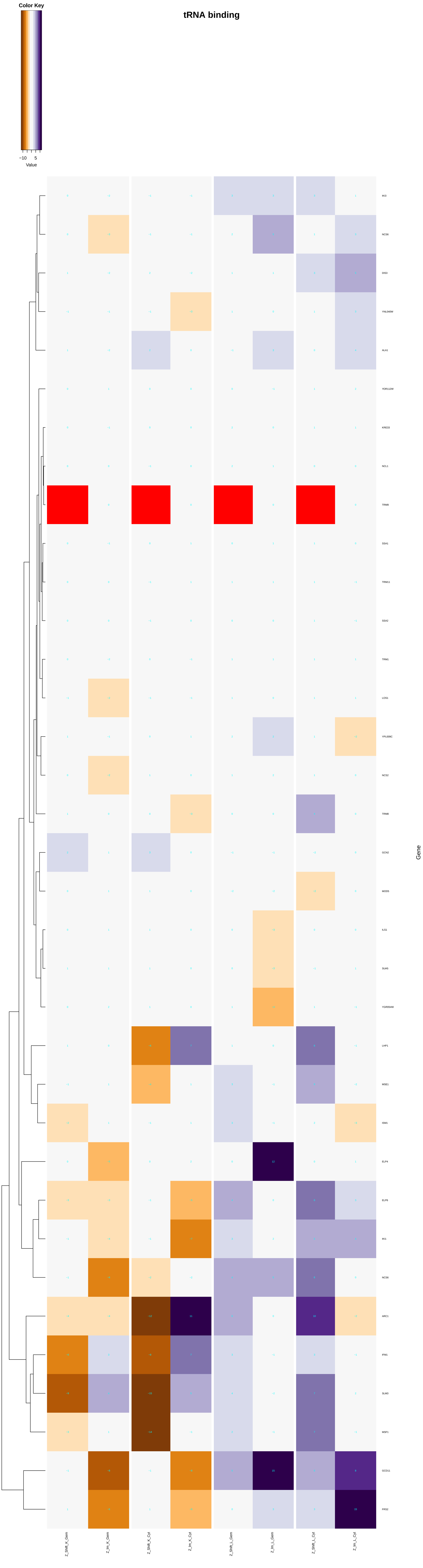
Color Key
−10
5
Value
tRNA binding
IKI3
NCS6
DIS3
YNL040W
ALA1
YOR112W
KRE33
NCL1
TRM9
SSA1
TRM11
SSA2
TRM1
LOS1
YPL009C
NCS2
TRM8
GCN2
MOD5
ILS1
SUA5
YGR054W
LHP1
MSE1
ISM1
ELP4
ELP6
IKI1
NCS6
ARC1
IFM1
SLM3
MSF1
GCD11
FRS2
Gene
Z_Shift_K_Gem
Z_Im_K_Gem
Z_Shift_K_Cyt
Z_Im_K_Cyt
Z_Shift_L_Gem
Z_Im_L_Gem
Z_Shift_L_Cyt
Z_Im_L_Cyt

RNA cap binding

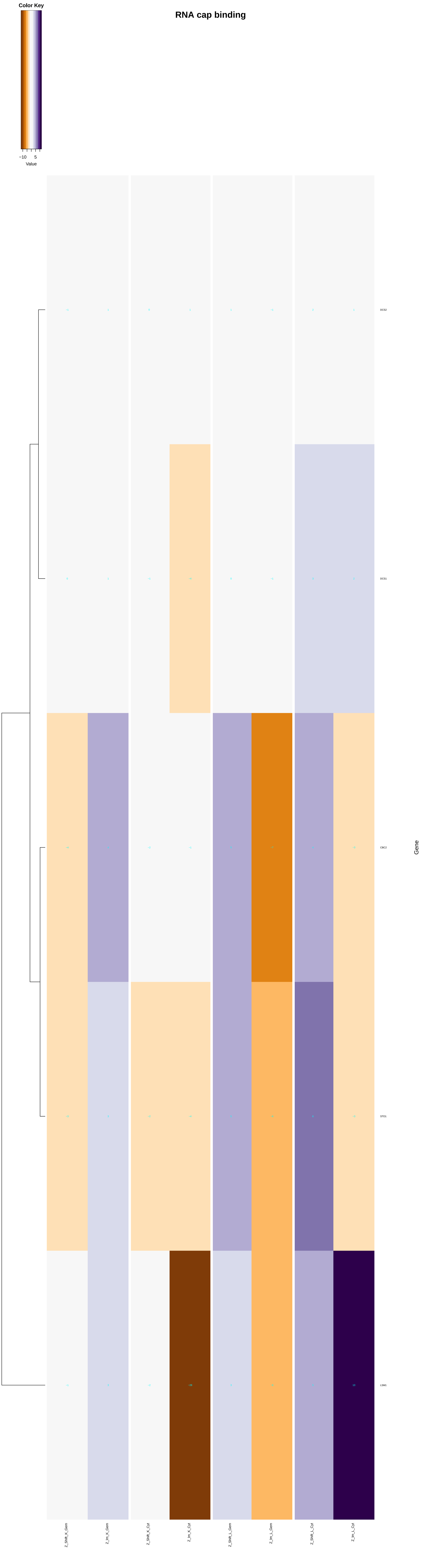

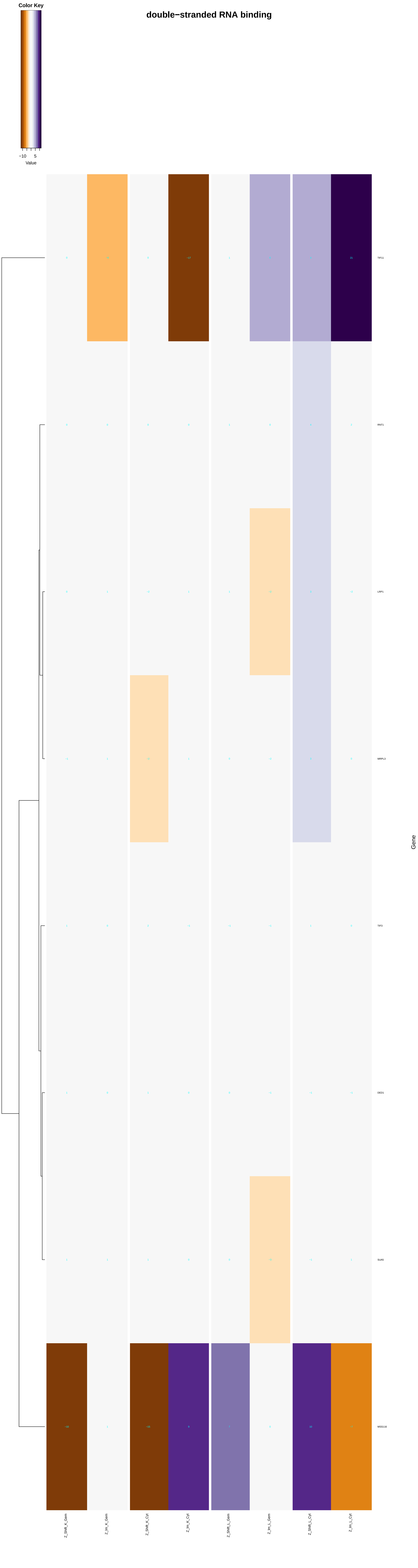

Color Key
−10
5
Value
double−stranded RNA binding
TIF11
RNT1
LRP1
MRPL3
TIF3
DED1
SUA5
MSS116
Gene
Z_Shift_K_Gem
Z_Int_K_Gem
Z_Shift_K_Cyt
Z_Int_K_Cyt
Z_Shift_L_Gem
Z_Int_L_Gem
Z_Shift_L_Cyt
Z_Int_L_Cyt

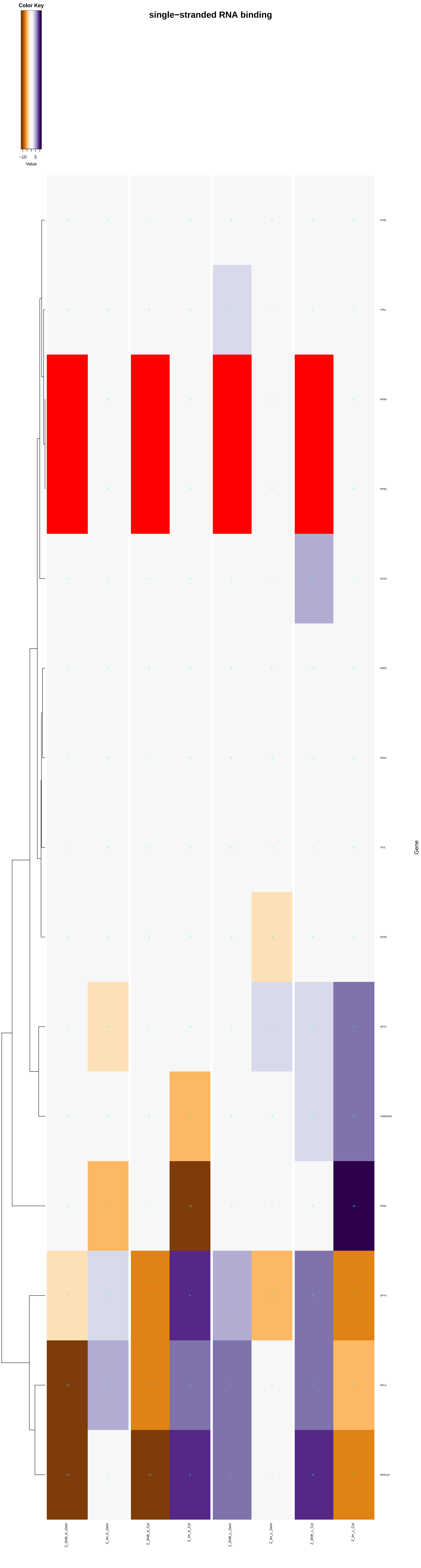
Color Key
−10
5
Value
single−stranded RNA binding
PUB1
TPA1
RPB4
RPB4
SGN1
RRP5
DED1
TIF3
MTR4
SPT5
YNR024W
RPB7
SPT4
NPL3
MSS116
Gene
Z_Shift_K_Gem
Z_Im_K_Gem
Z_Shift_K_Cyt
Z_Im_K_Cyt
Z_Shift_L_Gem
Z_Im_L_Gem
Z_Shift_L_Cyt
Z_Im_L_Cyt

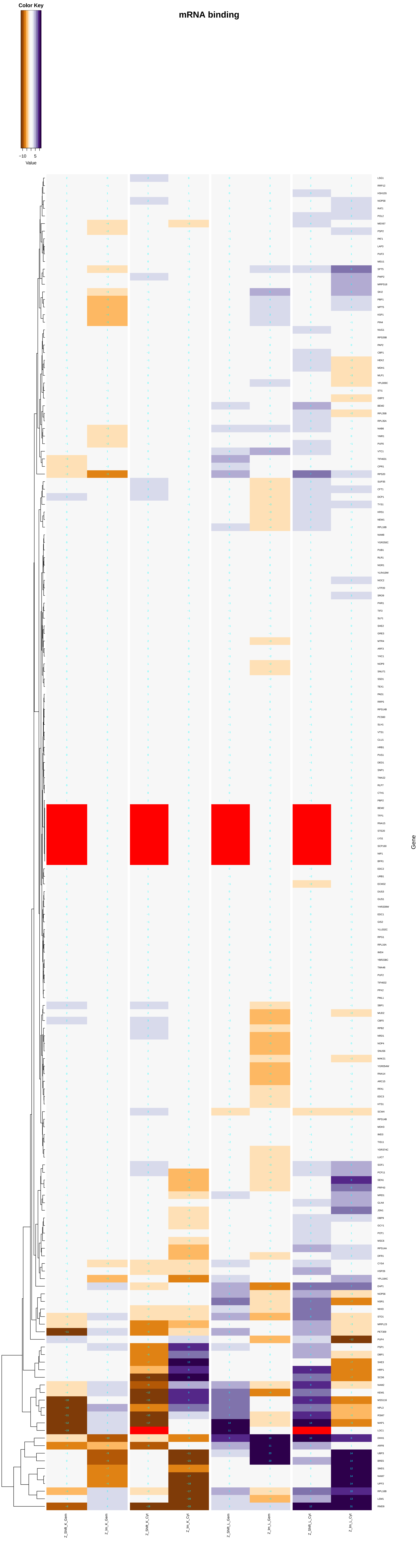
Color Key
Value
−10
5
mRNA binding
Gene
Z_Shift_X_Gem
Z_Im_X_Gem
Z_Shift_X_Cyt
Z_Im_X_Cyt
Z_Shift_L_Gem
Z_Im_L_Gem
Z_Shift_L_Cyt
Z_Im_L_Cyt

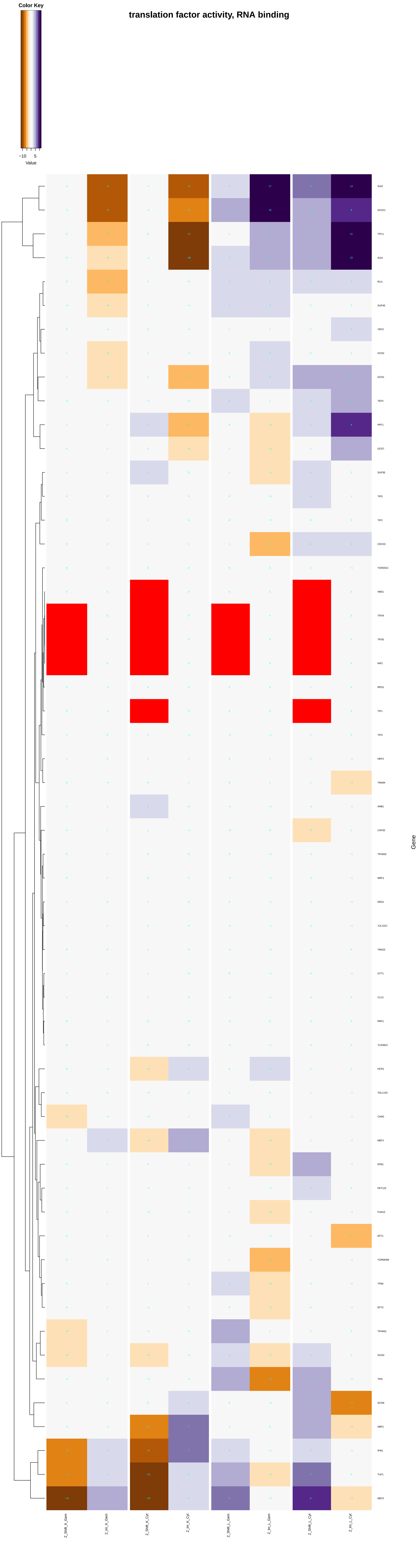

Color Key
Value
translation factor activity, RNA binding
Gene
SUI2
GCD11
TIF11
SUI3
RLI1
SUP45
YEF3
GCD2
GCD1
TEF4
PRT1
GCD7
SUP35
TIF5
TIF2
CDC33
YGR201C
HBS1
TIF34
TIF35
NIP1
RPG1
TIF1
TIF3
HEF3
TMA64
ANB1
CAF20
TIF4632
MRP1
DED1
YJL131C
TMA22
GIR2
CLU1
NMF1
YLR281C
HCR1
YDL114C
CAM1
MEF1
EFB1
PET122
FUN12
EFT1
YGR054W
TEF4
EFT2
TIF4631
GCN3
TIF6
GCD6
DBP1
IFM1
TUF1
MEF2
Z_Shift_K_Gem
Z_Int_K_Gem
Z_Shift_K_Cyt
Z_Int_K_Cyt
Z_Shift_L_Gem
Z_Int_L_Gem
Z_Shift_L_Cyt
Z_Int_L_Cyt

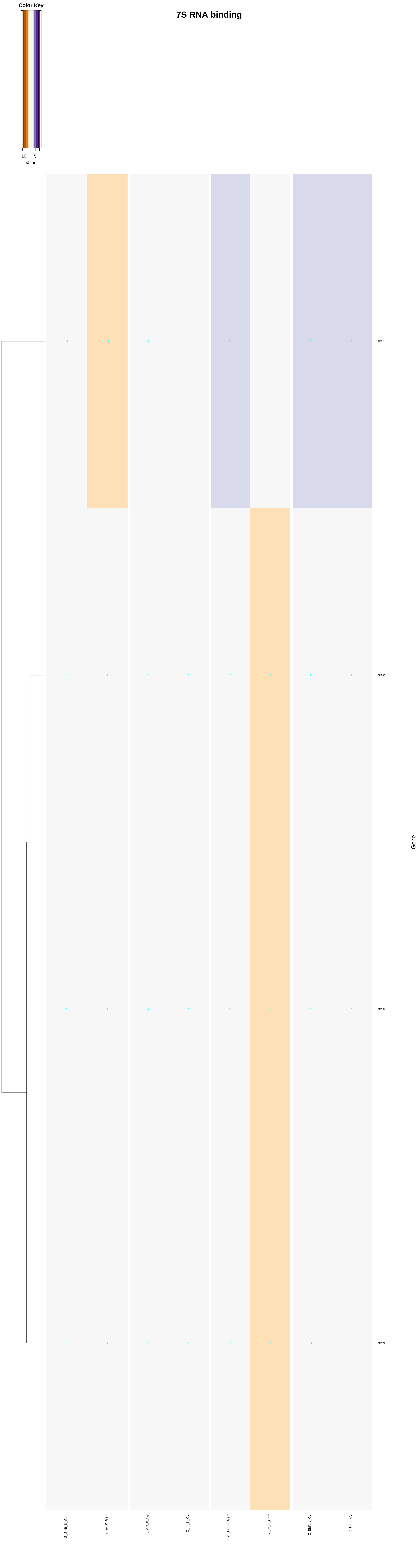

Color Key
−10
5
Value
7S RNA binding
RPF2
SRP68
SRP54
SRP72
Gene
Z_Shift_K_Gem
Z_Im_K_Gem
Z_Shift_K_Cyt
Z_Im_K_Cyt
Z_Shift_L_Gem
Z_Im_L_Gem
Z_Shift_L_Cyt
Z_Im_L_Cyt

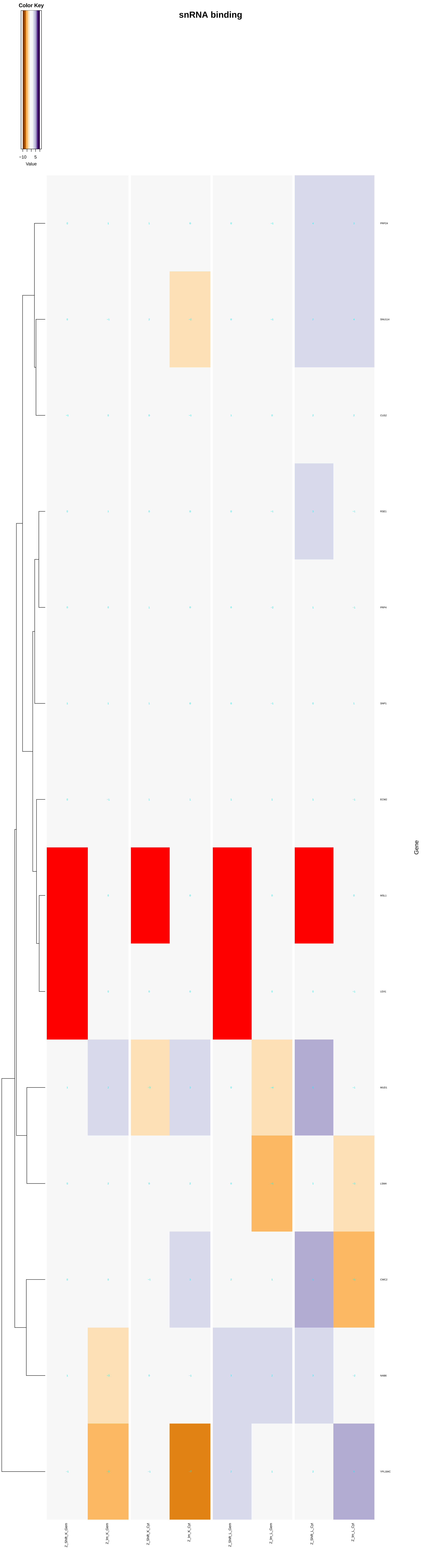

Color Key
−10
5
Value
snRNA binding
PRP24
SNU114
CUS2
RSE1
PRP4
SNP1
ECM2
MSL1
LEA1
MUD1
LSM4
CWC2
NAB6
YPL184C
Gene
Z_Shift_K_Gem
Z_Im_K_Gem
Z_Shift_K_Cyt
Z_Im_K_Cyt
Z_Shift_L_Gem
Z_Im_L_Gem
Z_Shift_L_Cyt
Z_Im_L_Cyt

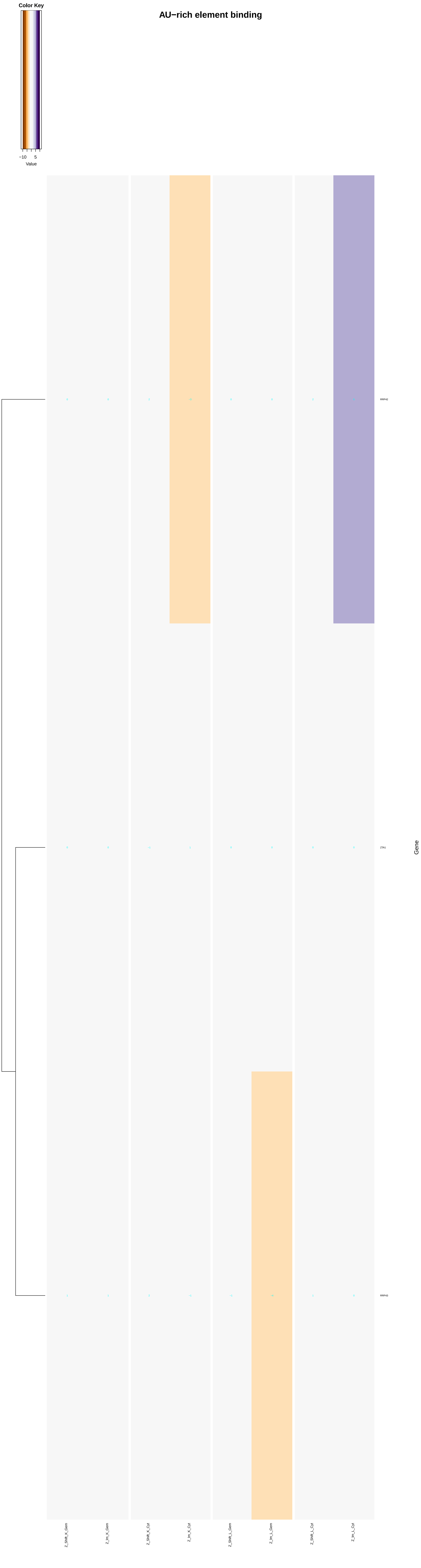
AU−rich element binding
Color Key
−10
5
Value
RRP42
ZTA1
RRP43
Gene
Z_Shift_K_Gem
Z_Im_K_Gem
Z_Shift_K_Cyt
Z_Im_K_Cyt
Z_Shift_L_Gem
Z_Im_L_Gem
Z_Shift_L_Cyt
Z_Im_L_Cyt

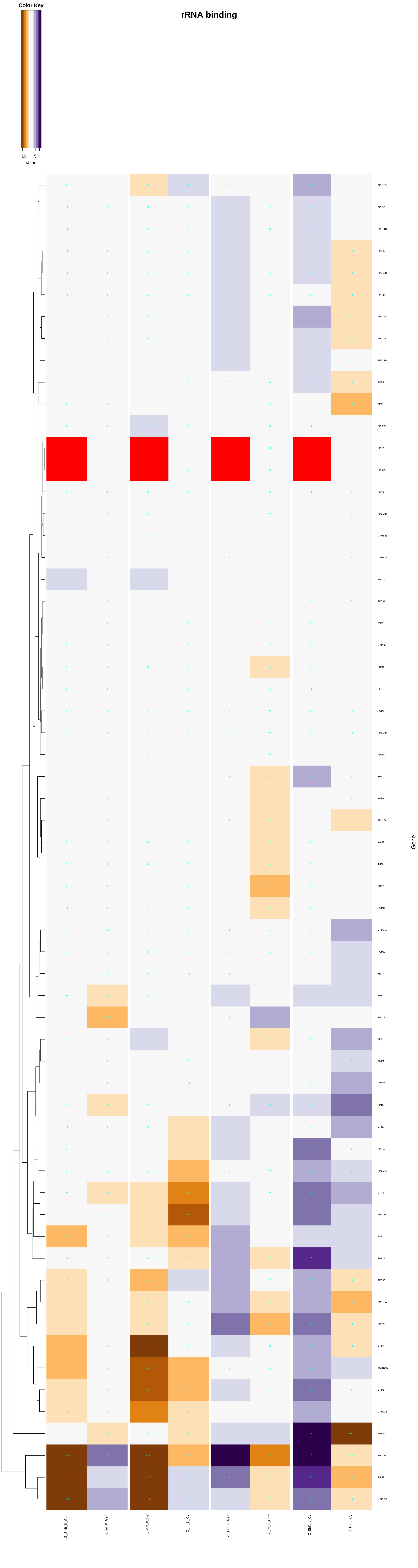

Color Key
Value
−10
5
rRNA binding
Gene
RPL11B
RPL3B
RPS11B
RPS4B
RPS18B
RPP2A
RPL37A
RPL37B
RPS11A
POP4
EFT1
RPL23B
RPS2
RPL37B
RRP5
RPS24B
MRPS28
MRPS17
RPL2A
RPS9A
SSF2
MRPL8
SNM1
RLP7
NOP6
RPS14B
RPL9A
BRX1
NOB1
RPL12A
NAM9
IMP4
POP6
NOP15
MRPS18
NOP53
YIF3
RRP2
RPL2B
ERB1
RMP1
UTP23
SPT5
MRD1
RPP1B
RPS14A
MRT4
RPL23A
SSF1
RPP1A
RPS9B
RPS18A
RPP2B
MRH4
YGR150C
MRP17
MRPL19
RPS4A
RPL12B
RSM7
MRPL38
Z_Shift_X_Gem
Z_Int_X_Gem
Z_Shift_X_Cyt
Z_Int_X_Cyt
Z_Shift_L_Gem
Z_Int_L_Gem
Z_Shift_L_Cyt
Z_Int_L_Cyt

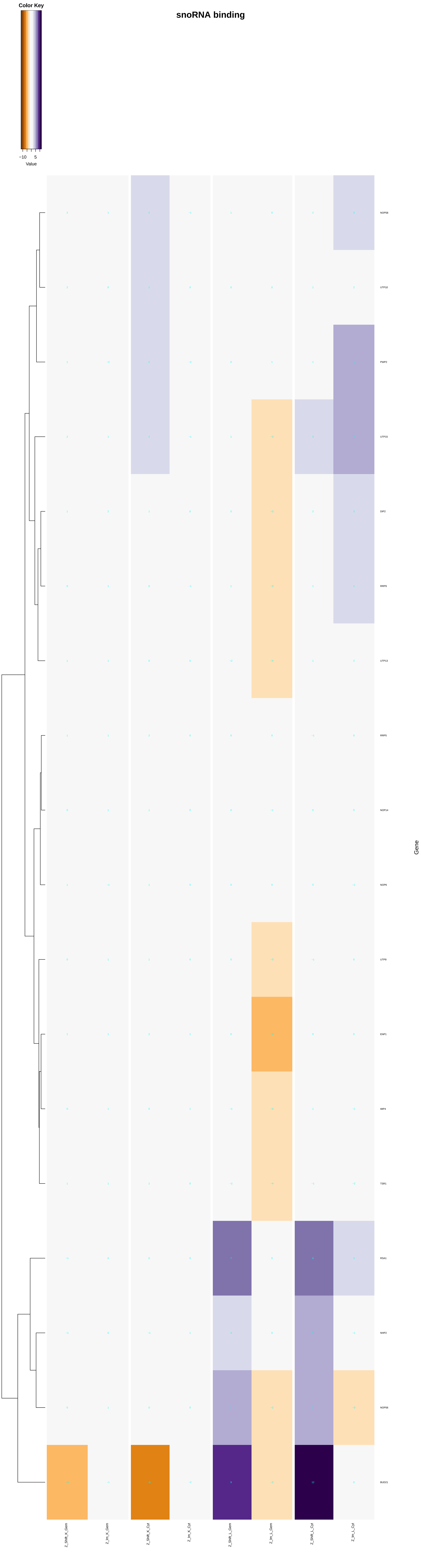
Color Key
−10
5
Value
snoRNA binding
NOP58
UTP10
PWP2
UTP15
DIP2
RRP9
UTP13
RRP5
NOP14
NOP6
UTP9
ENP1
IMP4
TSR1
RSA1
NHP2
NOP56
BUD21
Gene
Z_Shift_K_Gem
Z_Im_K_Gem
Z_Shift_K_Cyt
Z_Im_K_Cyt
Z_Shift_L_Gem
Z_Im_L_Gem
Z_Shift_L_Cyt
Z_Im_L_Cyt

pre−mRNA binding

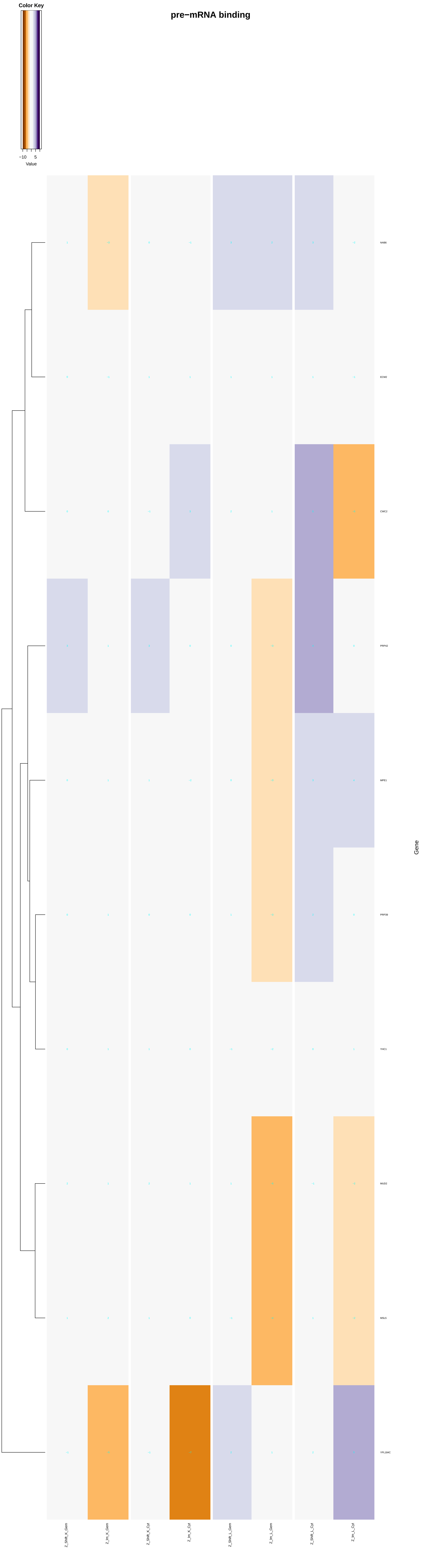

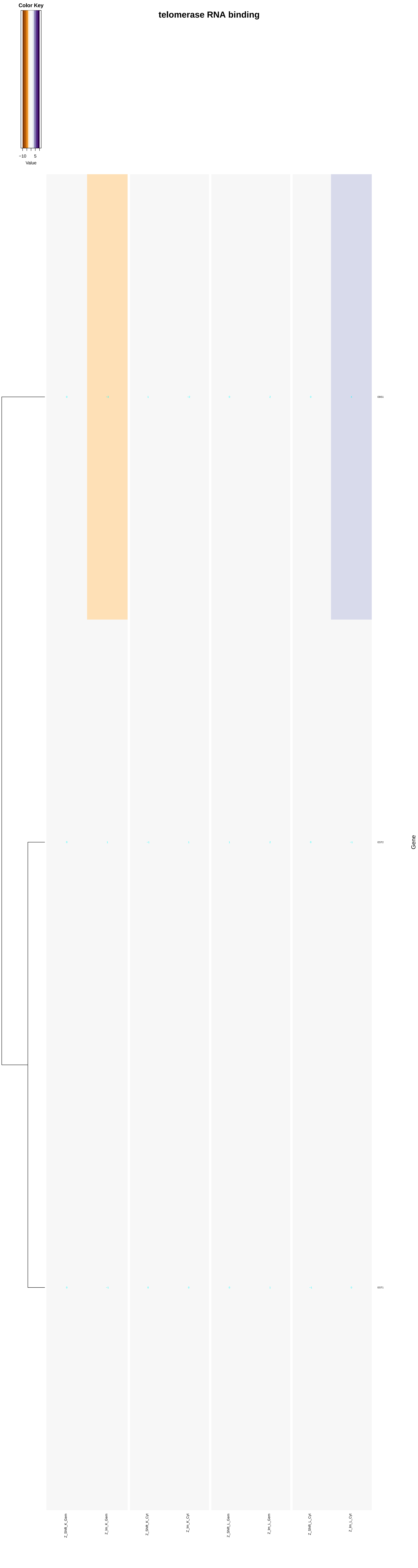
telomerase RNA binding
Color Key
−10
5
Value
EBS1
EST2
EST1
Gene
Z_Shift_K_Gem
Z_Im_K_Gem
Z_Shift_K_Cyt
Z_Im_K_Cyt
Z_Shift_L_Gem
Z_Im_L_Gem
Z_Shift_L_Cyt
Z_Im_L_Cyt

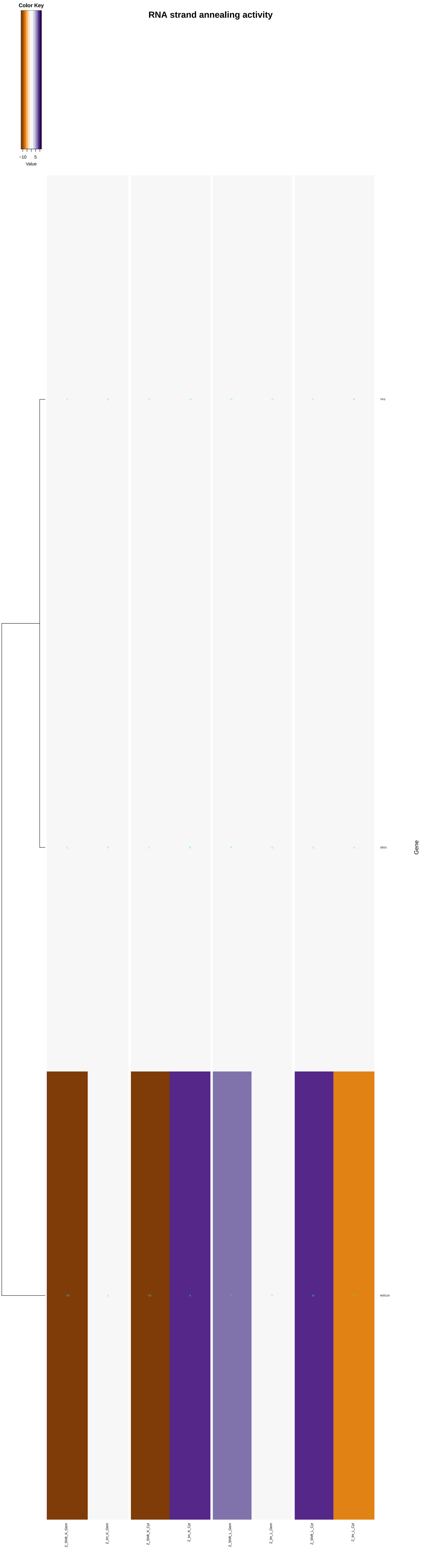

Color Key
−10
5
Value
RNA strand annealing activity
1
0
2
−1
−1
−1
1
0
TIF3
1
0
1
0
0
−1
−1
−1
DED1
−10
1
−15
9
7
0
10
−7
MSS116
Gene
Z_Shift_K_Gem
Z_Im_K_Gem
Z_Shift_K_Cyt
Z_Im_K_Cyt
Z_Shift_L_Gem
Z_Im_L_Gem
Z_Shift_L_Cyt
Z_Im_L_Cyt

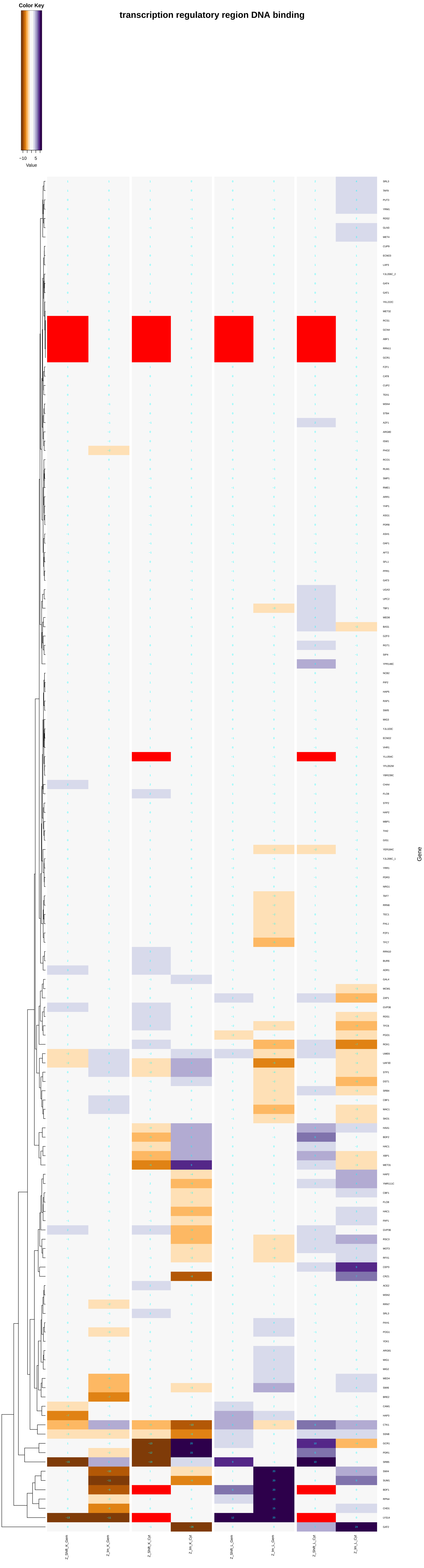
Color Key
−10
5
Value
transcription regulatory region DNA binding
Gene
Z_Shift_K_Gem
Z_Im_K_Gem
Z_Shift_K_Cyt
Z_Im_K_Cyt
Z_Shift_L_Gem
Z_Im_L_Gem
Z_Shift_L_Cyt
Z_Im_L_Cyt

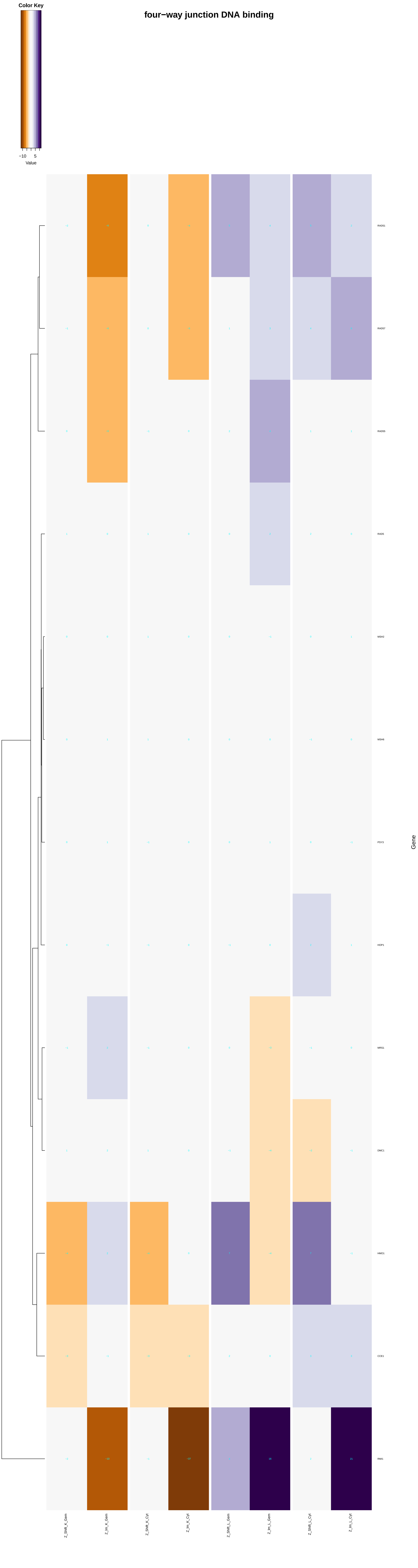
four−way junction DNA binding
Color Key
−10
5
Value
RAD51
RAD57
RAD55
RAD5
MSH2
MSH6
PSY3
HOP1
MRS1
DMC1
HMO1
CCE1
RMI1
Gene
Z_Shift_K_Gem
Z_Im_K_Gem
Z_Shift_K_Cyt
Z_Im_K_Cyt
Z_Shift_L_Gem
Z_Im_L_Gem
Z_Shift_L_Cyt
Z_Im_L_Cyt

Y−form DNA binding

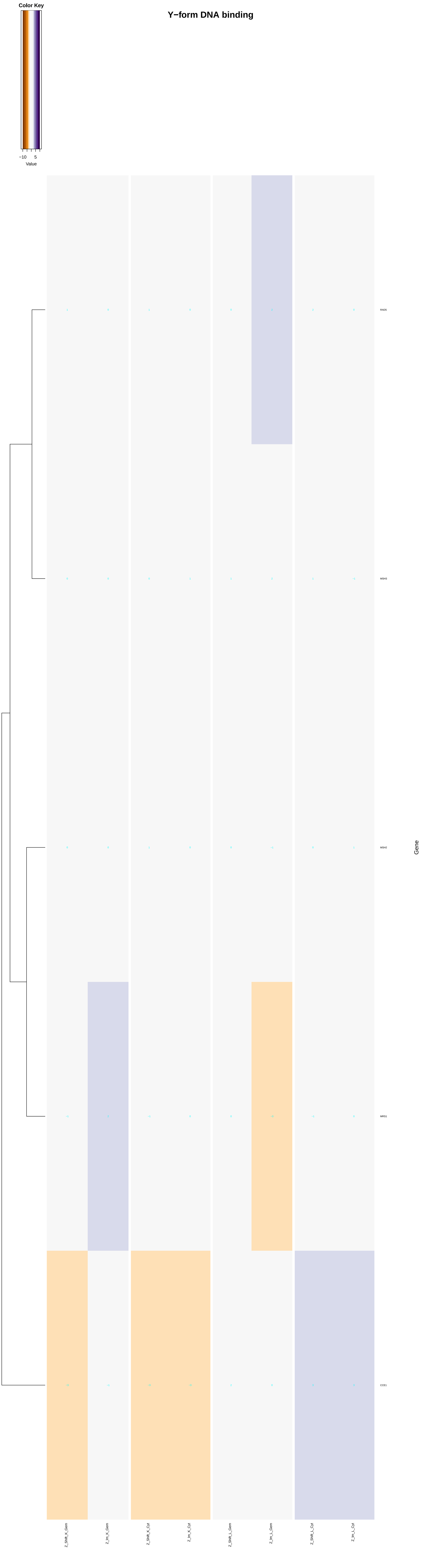

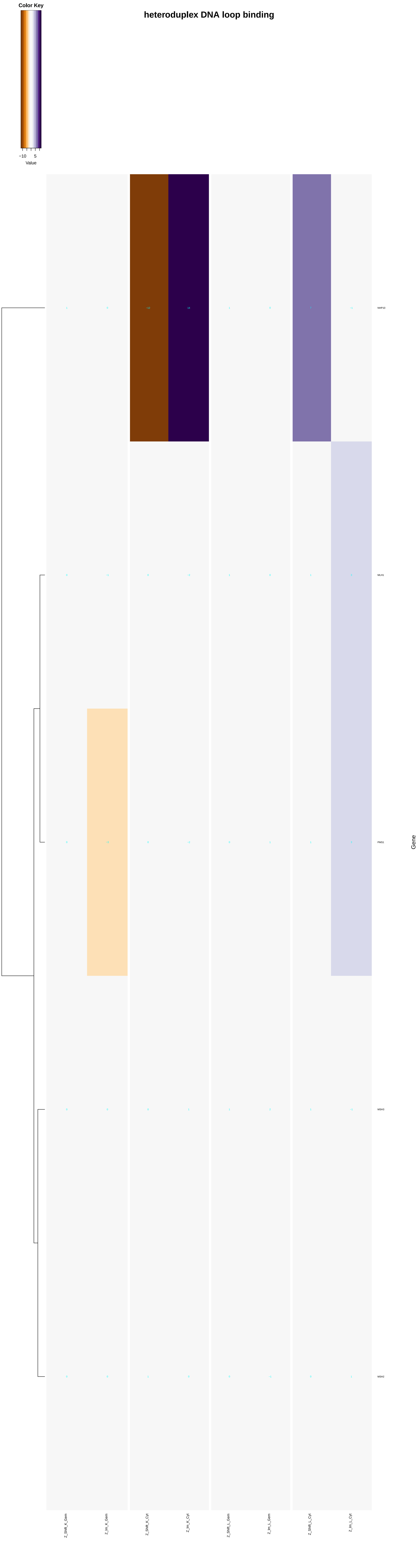

heteroduplex DNA loop binding
Color Key
−10
5
Value
NHP10
MLH1
PMS1
MSH3
MSH2
Gene
Z_Shift_K_Gem
Z_Im_K_Gem
Z_Shift_K_Cyt
Z_Im_K_Cyt
Z_Shift_L_Gem
Z_Im_L_Gem
Z_Shift_L_Cyt
Z_Im_L_Cyt

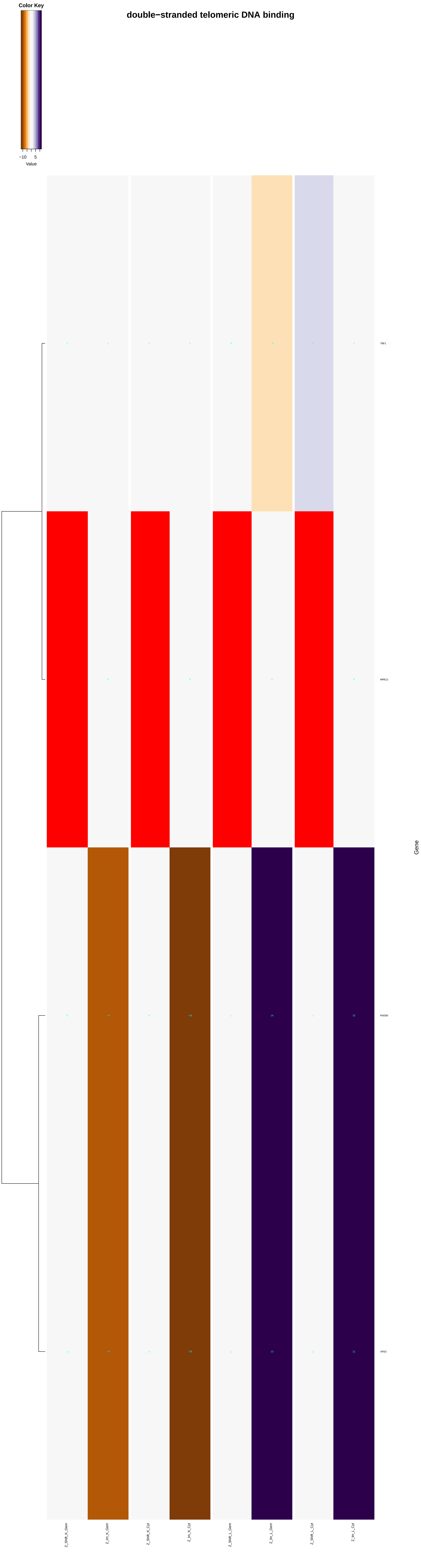
double−stranded telomeric DNA binding
Color Key
−10
5
Value
2
1
1
1
0
−3
2
1
TBF1
0
0
0
0
MRE11
0
−9
0
−40
1
14
1
32
RAD50
−1
−9
−1
−39
1
13
2
21
XRS2
Gene
Z_Shift_K_Gem
Z_Im_K_Gem
Z_Shift_K_Cyt
Z_Im_K_Cyt
Z_Shift_L_Gem
Z_Im_L_Gem
Z_Shift_L_Cyt
Z_Im_L_Cyt

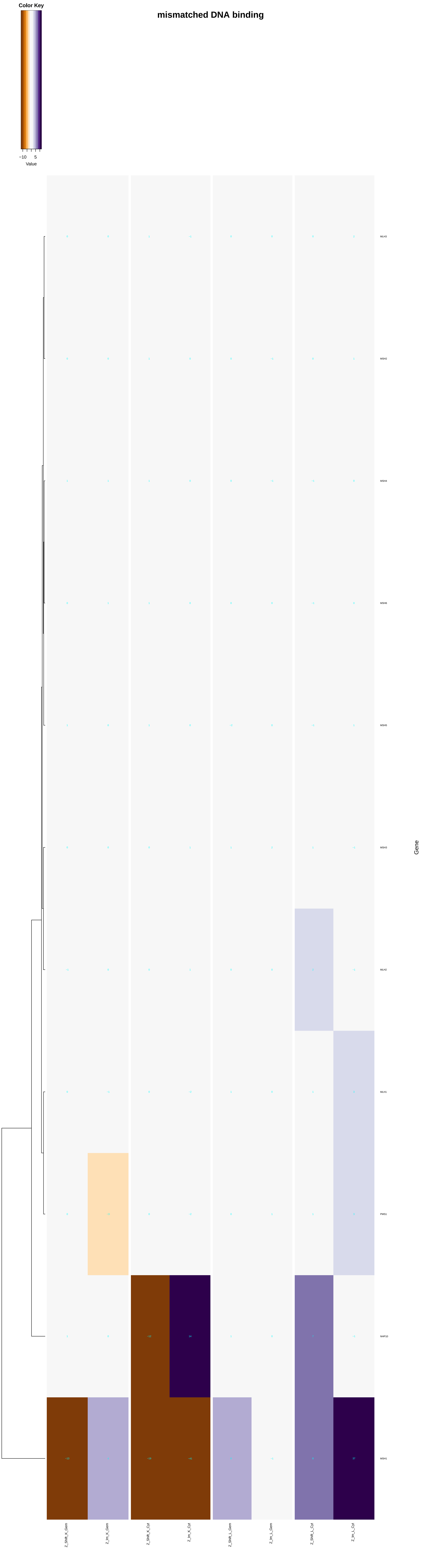

mismatched DNA binding
Color Key
−10
5
Value
MLH3
MSH2
MSH4
MSH6
MSH5
MSH3
MLH2
MLH1
PMS1
NHP10
MSH1
Gene
Z_Shift_K_Gem
Z_Im_K_Gem
Z_Shift_K_Cyt
Z_Im_K_Cyt
Z_Shift_L_Gem
Z_Im_L_Gem
Z_Shift_L_Cyt
Z_Im_L_Cyt

sequence−specific double−stranded DNA binding

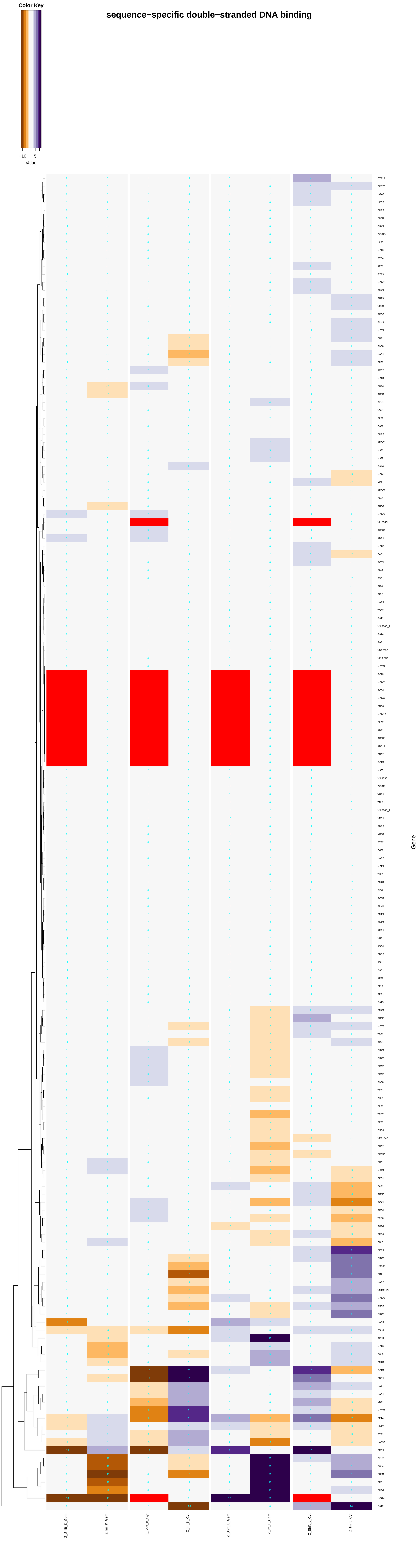

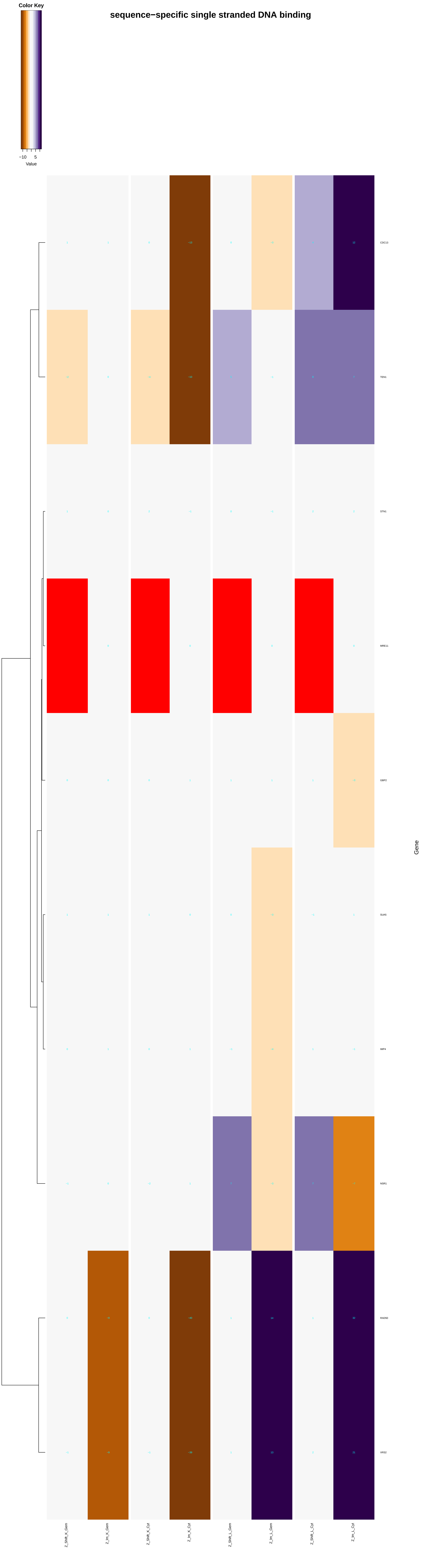

Color Key
−10
5
Value
sequence−specific single stranded DNA binding
CDC13
TEN1
STN1
MRE11
GBP2
SUA5
IMP4
NSR1
RAD50
XRS2
Gene
Z_Shift_K_Gem
Z_lm_K_Gem
Z_Shift_K_Cyt
Z_lm_K_Cyt
Z_Shift_L_Gem
Z_lm_L_Gem
Z_Shift_L_Cyt
Z_lm_L_Cyt

nucleosomal DNA binding

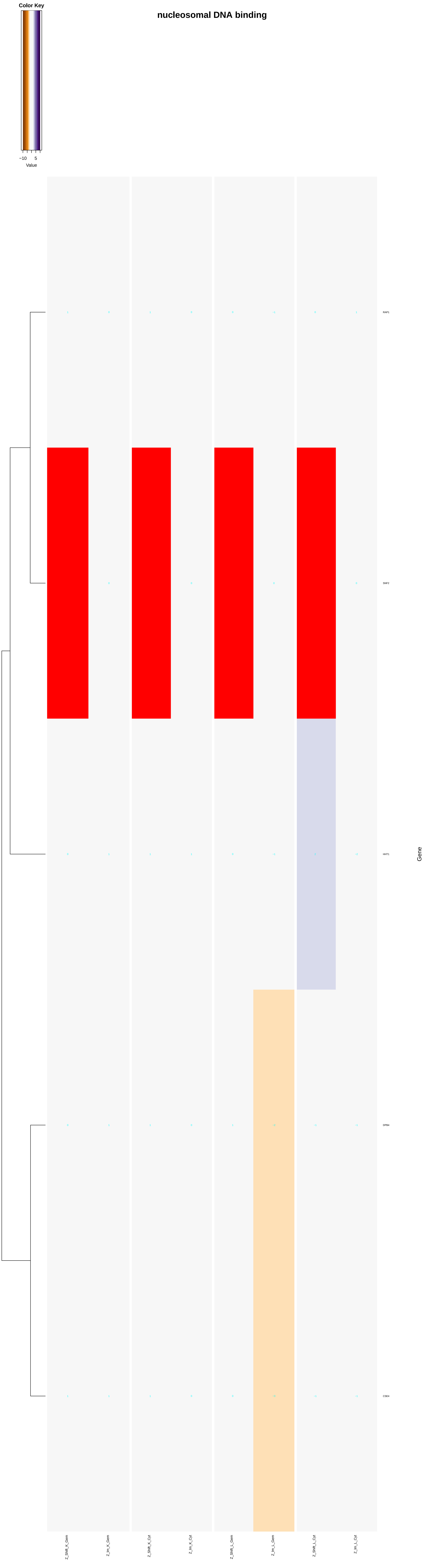

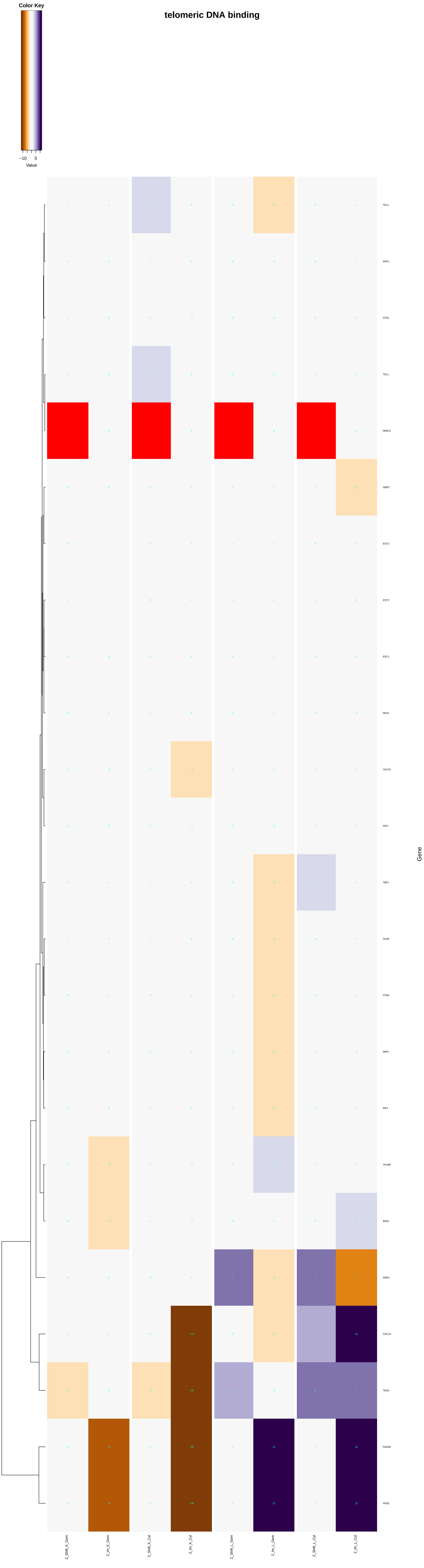
Color Key
−10
5
Value
telomeric DNA binding
TEL2
RAP1
STN1
TEL1
MRE11
GBP2
EST2
EST3
EST1
NDJ1
YKU70
RIF2
TBF1
SUA5
STM1
IMP4
RIF1
YKU80
EBS1
NSR1
CDC13
TEN1
RAD50
XRS2
Gene
Z_Shift_K_Gem
Z_Im_K_Gem
Z_Shift_K_Cyt
Z_Im_K_Cyt
Z_Shift_L_Gem
Z_Im_L_Gem
Z_Shift_L_Cyt
Z_Im_L_Cyt

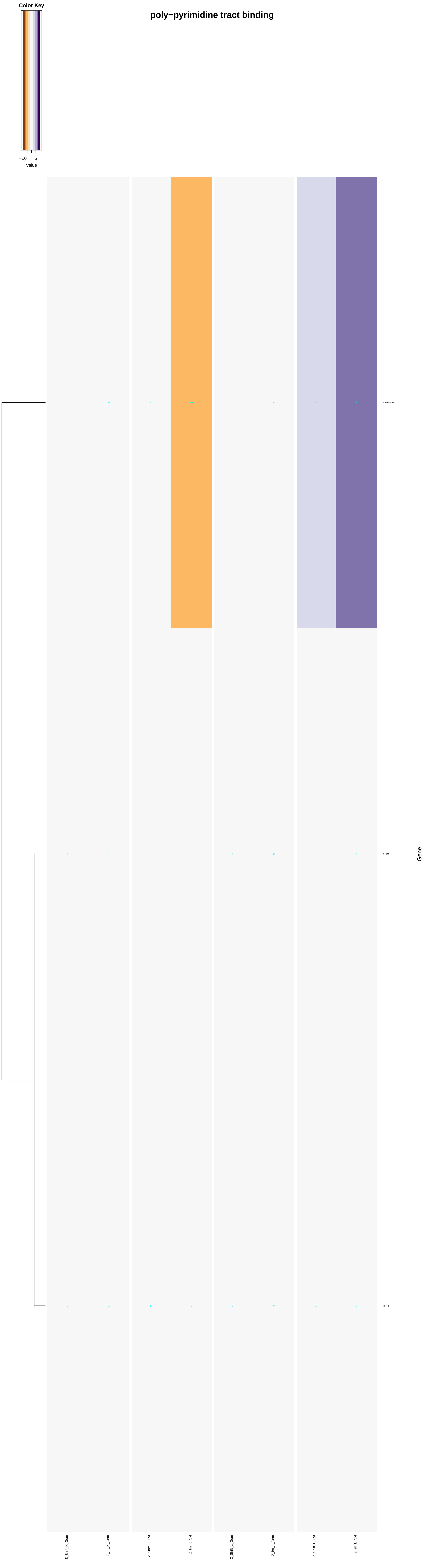
Color Key
−10
5
Value
poly−pyrimidine tract binding
YNR024W
PUB1
RRP5
Gene
Z_Shift_K_Gem
Z_Im_K_Gem
Z_Shift_K_Cyt
Z_Im_K_Cyt
Z_Shift_L_Gem
Z_Im_L_Gem
Z_Shift_L_Cyt
Z_Im_L_Cyt

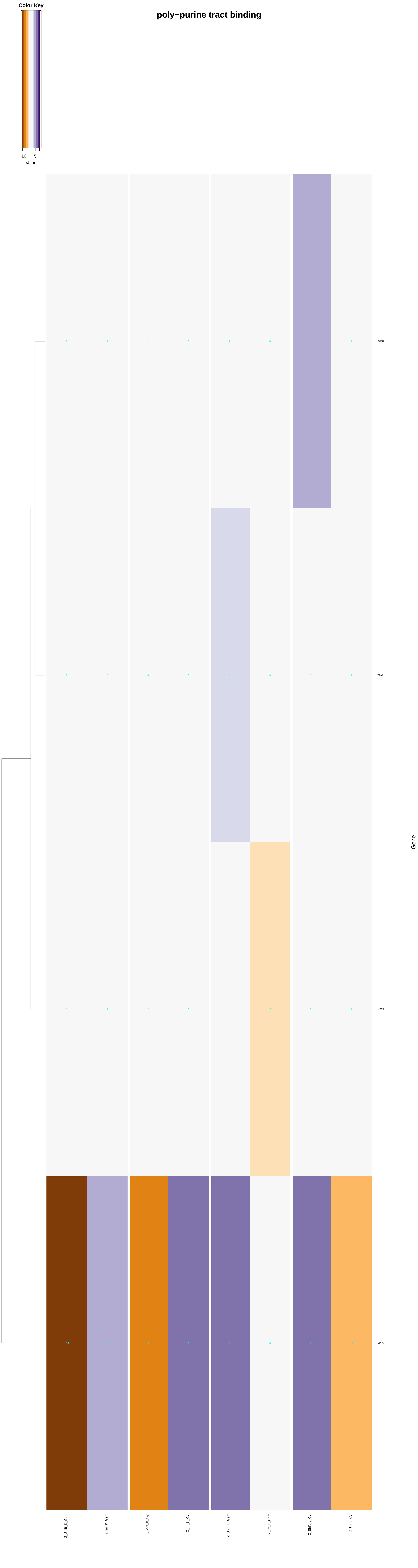
poly−purine tract binding
Color Key
−10
5
Value
SGN1
TPA1
MTR4
NPL3
Z_Shift_K_Gem
Z_Int_K_Gem
Z_Shift_K_Cyt
Z_Int_K_Cyt
Z_Shift_L_Gem
Z_Int_L_Gem
Z_Shift_L_Cyt
Z_Int_L_Cyt
Gene

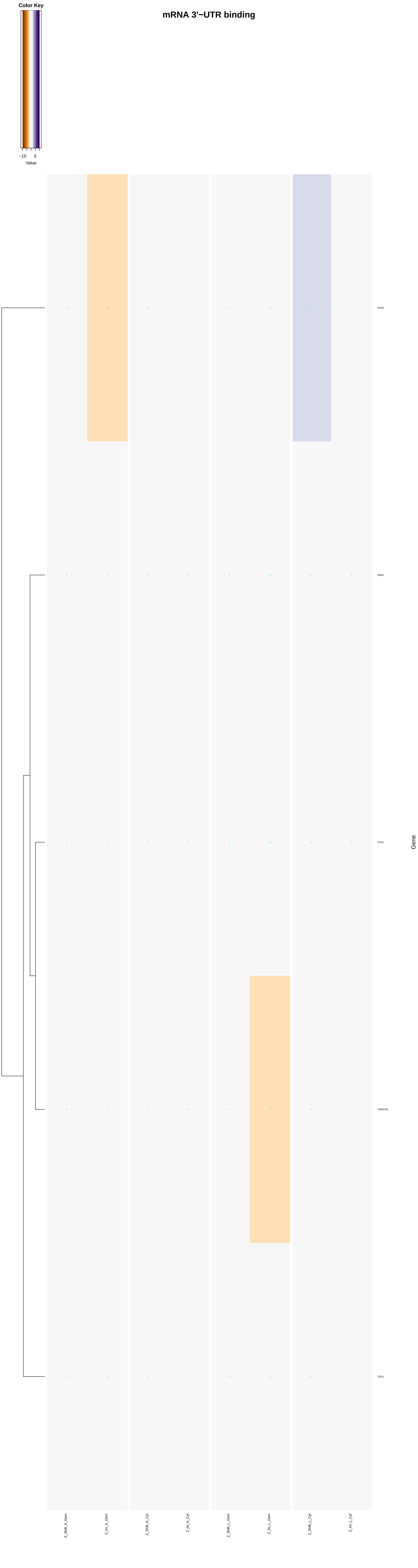
mRNA 3'−UTR binding
Color Key
−10
5
Value
PUF6
SSD1
CTH1
YDR374C
TIS11
Gene
Z_Shift_K_Gem
Z_Im_K_Gem
Z_Shift_K_Cyt
Z_Im_K_Cyt
Z_Shift_L_Gem
Z_Im_L_Gem
Z_Shift_L_Cyt
Z_Im_L_Cyt

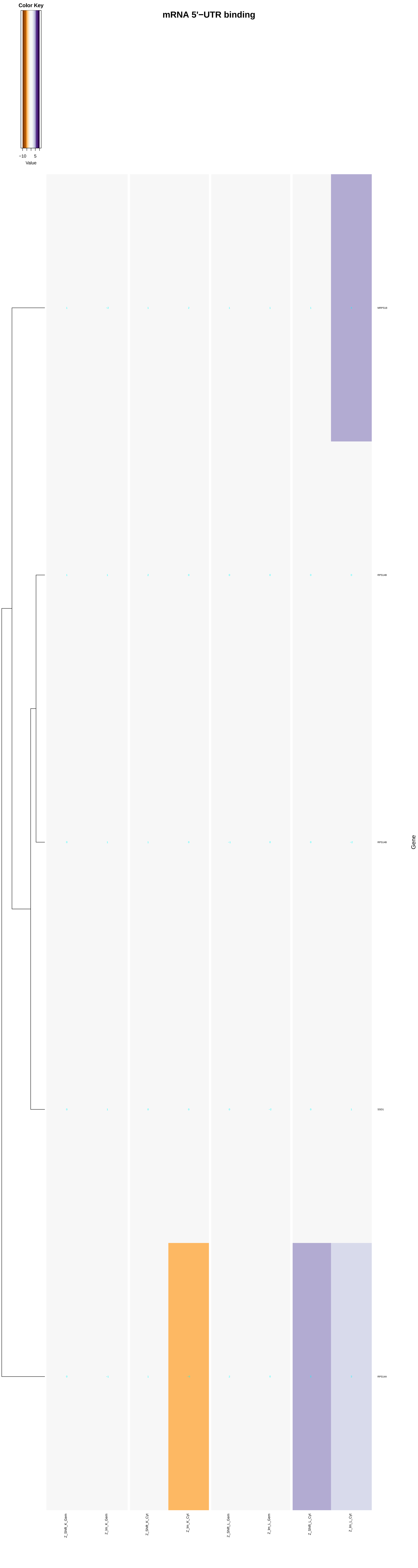
Color Key
−10
5
Value
mRNA 5′−UTR binding
MRPS18
RPS14B
RPS14B
SSD1
RPS14A
Gene
Z_Shift_K_Gem
Z_Int_K_Gem
Z_Shift_K_Cyt
Z_Int_K_Cyt
Z_Shift_L_Gem
Z_Int_L_Gem
Z_Shift_L_Cyt
Z_Int_L_Cyt

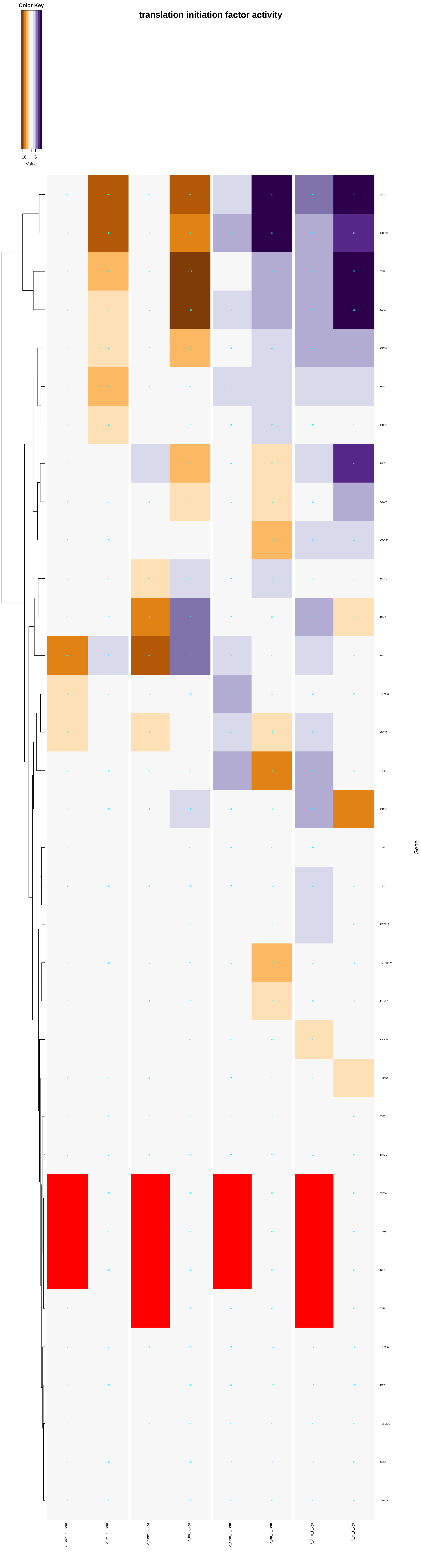
translation initiation factor activity
Color Key
−10
5
Value
SUI2
GCD11
TIF11
SUI1
GCD1
RLI1
GCD2
PRT1
GCD7
CDC33
HCR1
DBP1
IFM1
TIF4631
GCN3
TIF6
GCD6
TIF2
TIF5
PET122
YGR054W
FUN12
CAF20
TMA64
TIF3
RPG1
TIF34
TIF35
NIP1
TIF1
TIF4632
DED1
YJL131C
CLU1
TMA22
Gene
Z_Shift_K_Gem
Z_Im_K_Gem
Z_Shift_K_Cyt
Z_Im_K_Cyt
Z_Shift_L_Gem
Z_Im_L_Gem
Z_Shift_L_Cyt
Z_Im_L_Cyt

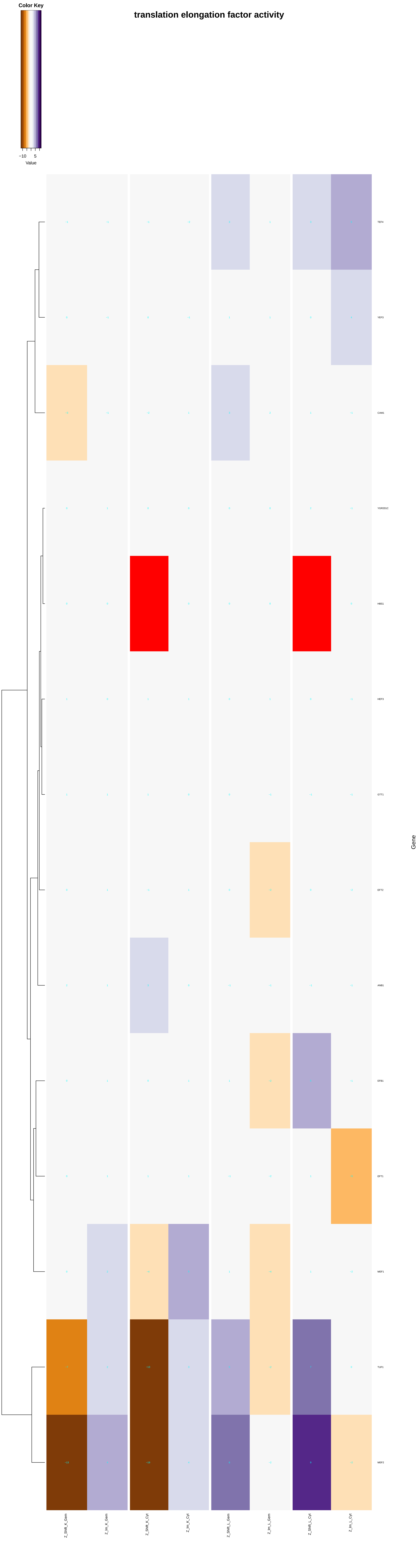
Color Key
−10
5
Value
translation elongation factor activity
TEF4
YEF3
CAM1
YGR201C
HBS1
HEF3
GTT1
EFT2
ANB1
EFB1
EFT1
MEF1
TUF1
MEF2
Gene
Z_Shift_K_Gem
Z_Int_K_Gem
Z_Shift_K_Cyt
Z_Int_K_Cyt
Z_Shift_L_Gem
Z_Int_L_Gem
Z_Shift_L_Cyt
Z_Int_L_Cyt

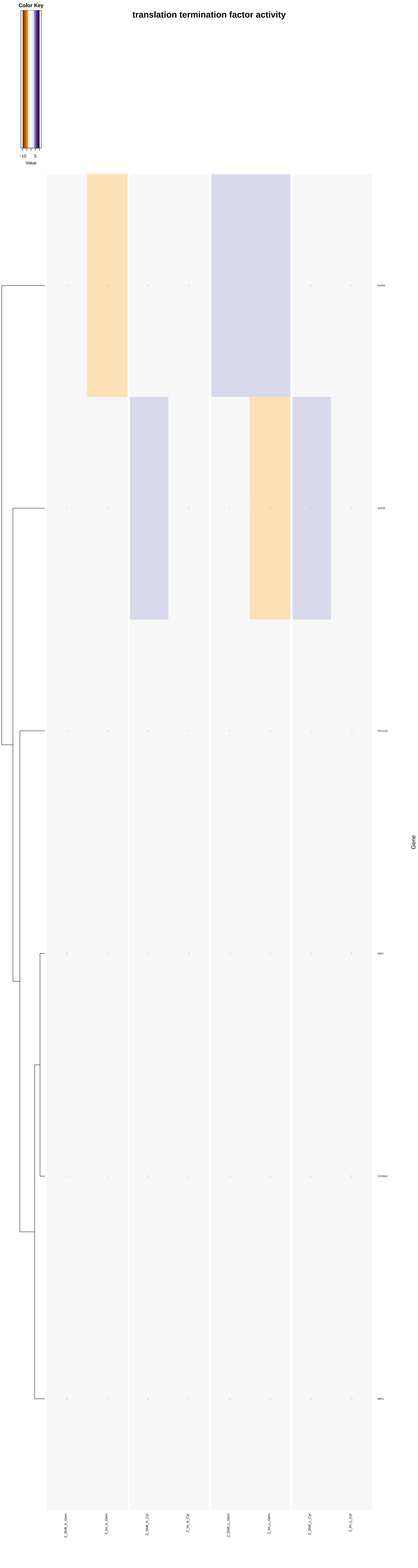
translation termination factor activity
Color Key
−10
5
Value
SUP45
SUP35
YOL114C
RRF1
YLR281C
MRF1
Gene
Z_Shift_K_Gem
Z_Im_K_Gem
Z_Shift_K_Cyt
Z_Im_K_Cyt
Z_Shift_L_Gem
Z_Im_L_Gem
Z_Shift_L_Cyt
Z_Im_L_Cyt

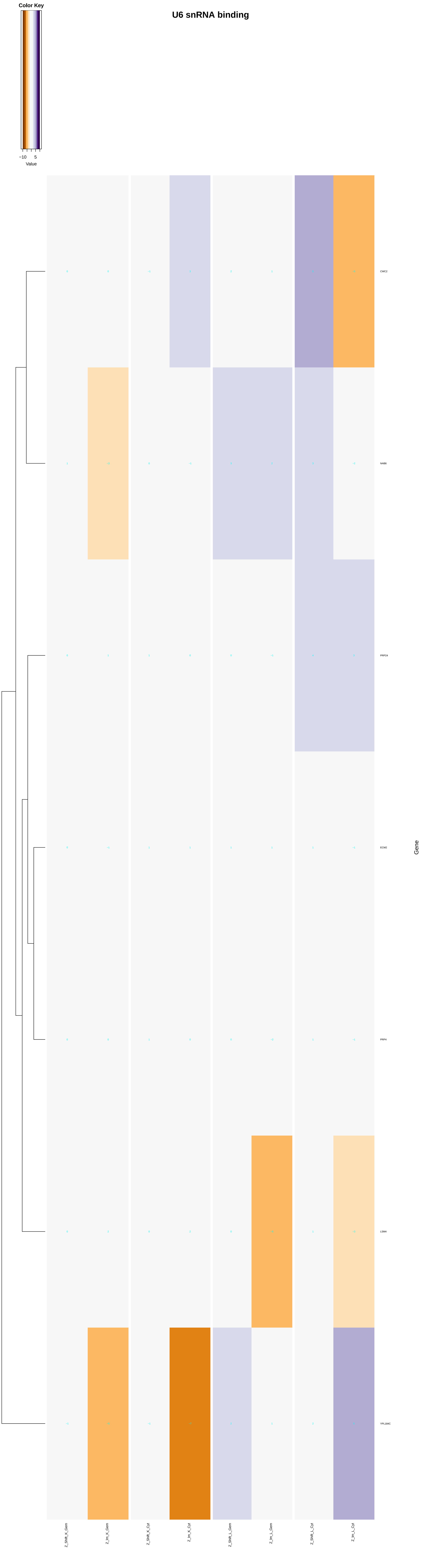

Color Key
−10
5
Value
U6 snRNA binding
CWC2
NAB6
PRP24
ECM2
PRP4
LSM4
YPL184C
Gene
Z_Shift_K_Gem
Z_Im_K_Gem
Z_Shift_K_Cyt
Z_Im_K_Cyt
Z_Shift_L_Gem
Z_Im_L_Gem
Z_Shift_L_Cyt
Z_Im_L_Cyt

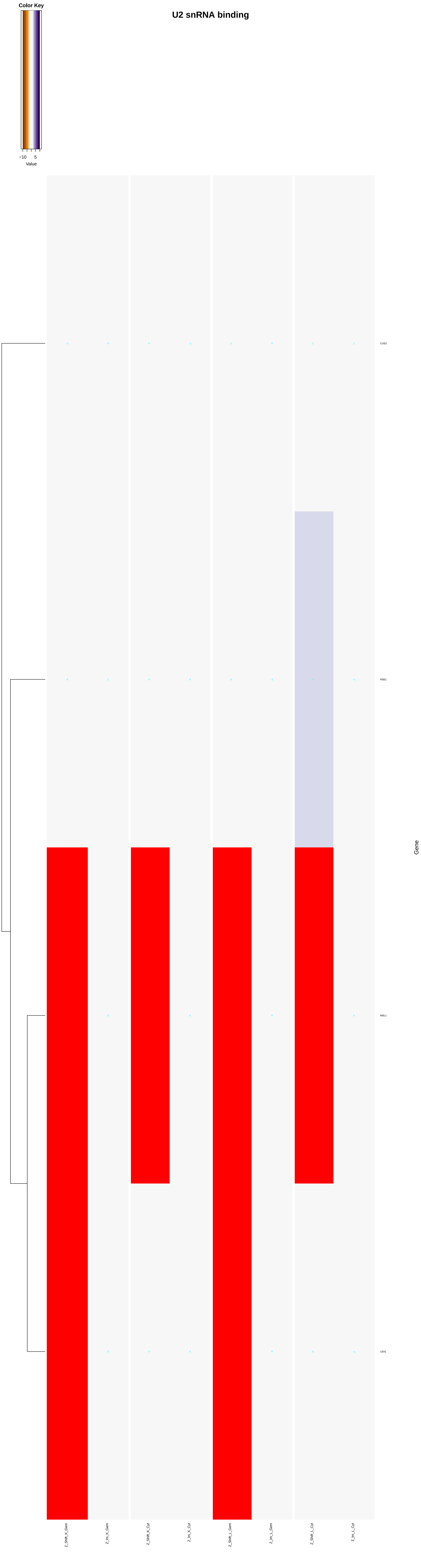

Color Key
−10
5
Value
U2 snRNA binding
CUS2
RSE1
MSL1
LEA1
Gene
Z_Shift_K_Gem
Z_Im_K_Gem
Z_Shift_K_Cyt
Z_Im_K_Cyt
Z_Shift_L_Gem
Z_Im_L_Gem
Z_Shift_L_Cyt
Z_Im_L_Cyt

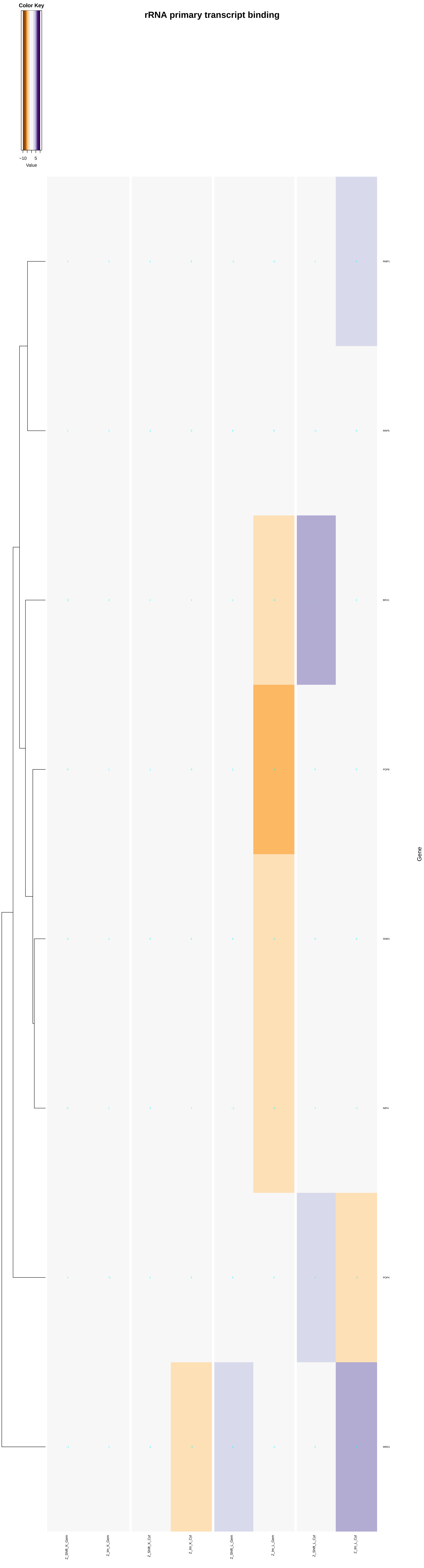

rRNA primary transcript binding
Color Key
−10
5
Value
RMP1
RRP5
BRX1
POP6
SNM1
IMP4
POP4
MRD1
Gene
Z_Shift_K_Gem
Z_Im_K_Gem
Z_Shift_K_Cyt
Z_Im_K_Cyt
Z_Shift_L_Gem
Z_Im_L_Gem
Z_Shift_L_Cyt
Z_Im_L_Cyt

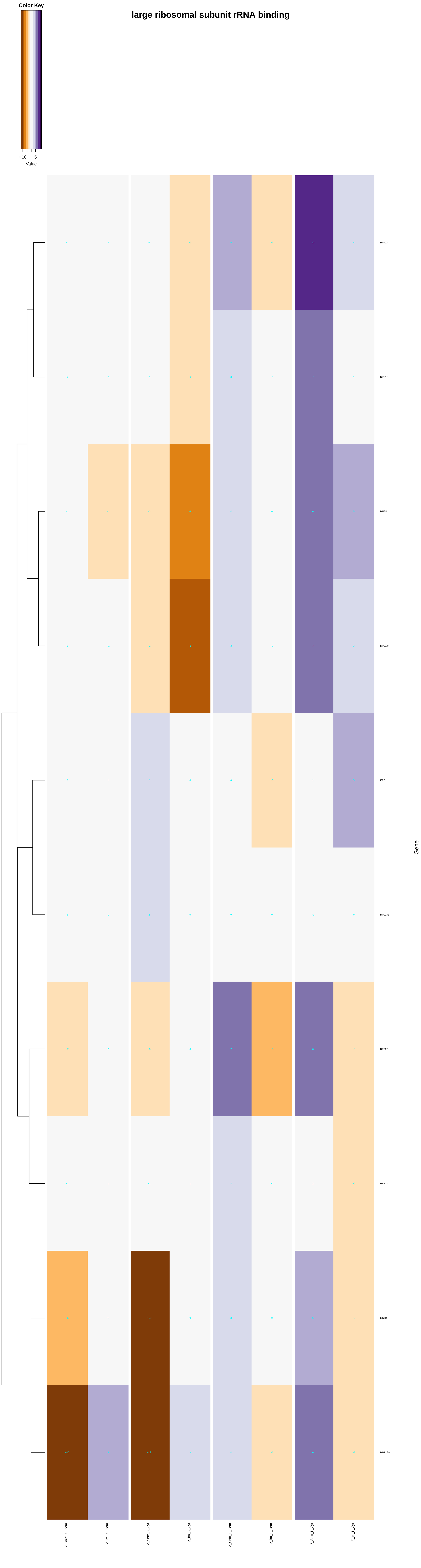
large ribosomal subunit rRNA binding
Color Key
−10
5
Value
RPP1A
RPP1B
MRT4
RPL23A
ERB1
RPL23B
RPP2B
RPP2A
MRH4
MRPL38
Gene
Z_Shift_K_Gem
Z_Im_K_Gem
Z_Shift_K_Cyt
Z_Im_K_Cyt
Z_Shift_L_Gem
Z_Im_L_Gem
Z_Shift_L_Cyt
Z_Im_L_Cyt

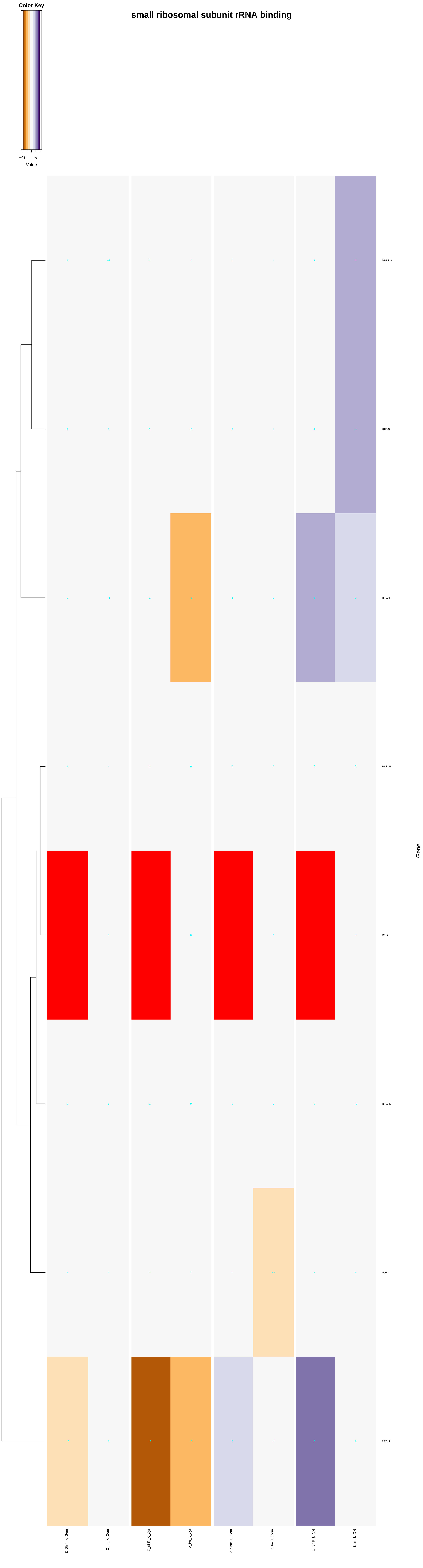

small ribosomal subunit rRNA binding
Color Key
−10
5
Value
MRPS18
UTP23
RPS14A
RPS14B
RPS2
RPS14B
NOB1
MRP17
Gene
Z_Shift_K_Gem
Z_Im_K_Gem
Z_Shift_K_Cyt
Z_Im_K_Cyt
Z_Shift_L_Gem
Z_Im_L_Gem
Z_Shift_L_Cyt
Z_Im_L_Cyt

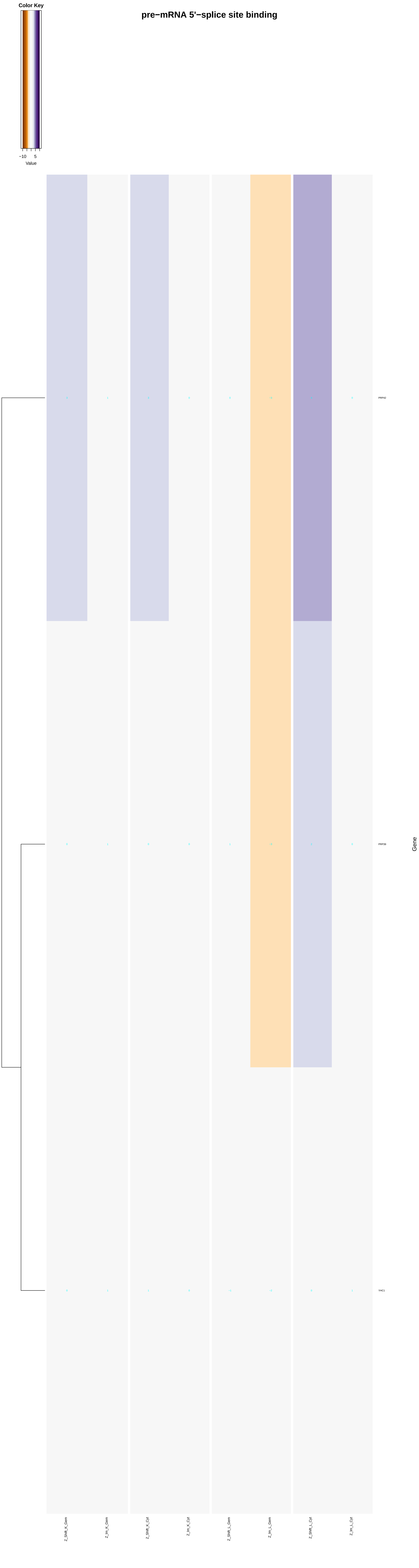

Color Key
−10
5
Value
pre−mRNA 5'−splice site binding
PRP42
PRP39
YHC1
Gene
Z_Shift_K_Gem
Z_Im_K_Gem
Z_Shift_K_Cyt
Z_Im_K_Cyt
Z_Shift_L_Gem
Z_Im_L_Gem
Z_Shift_L_Cyt
Z_Im_L_Cyt

transcription regulatory region sequence−specific DNA binding

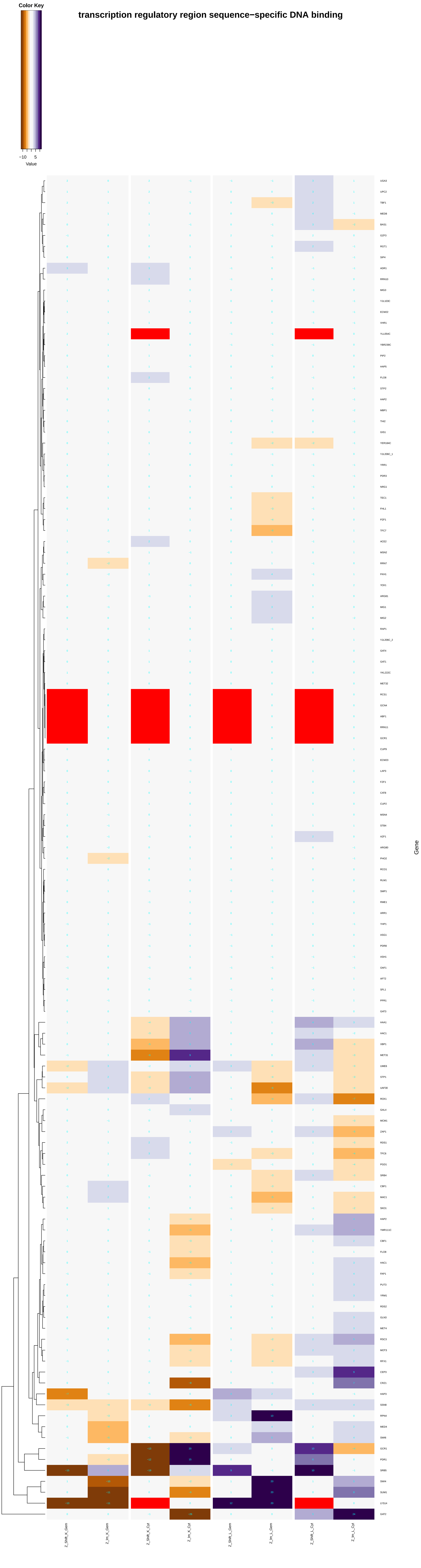

RNA polymerase II regulatory region DNA binding

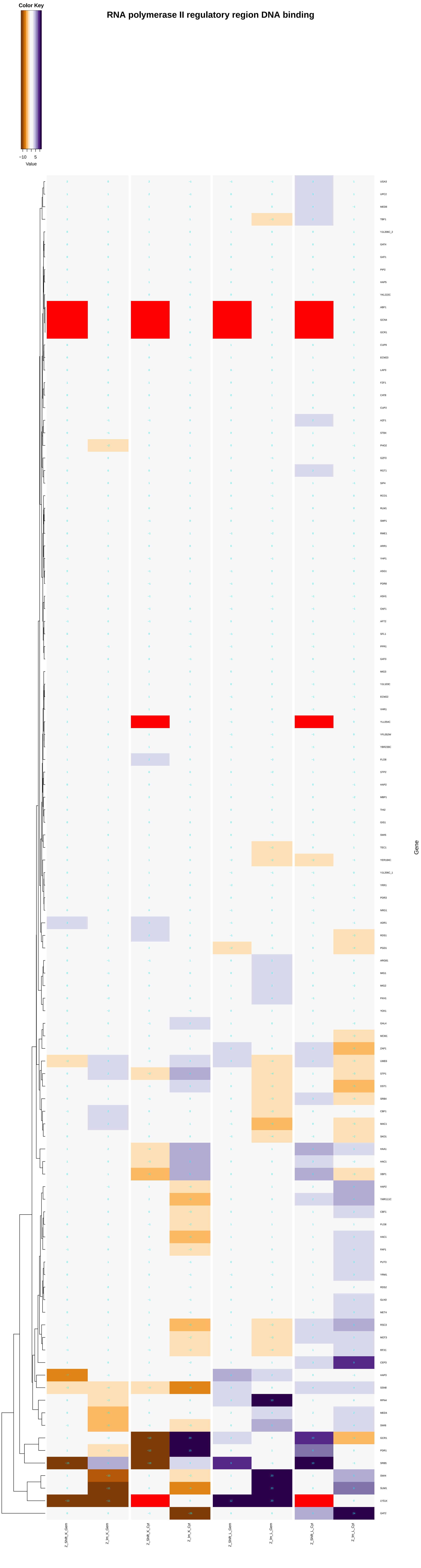

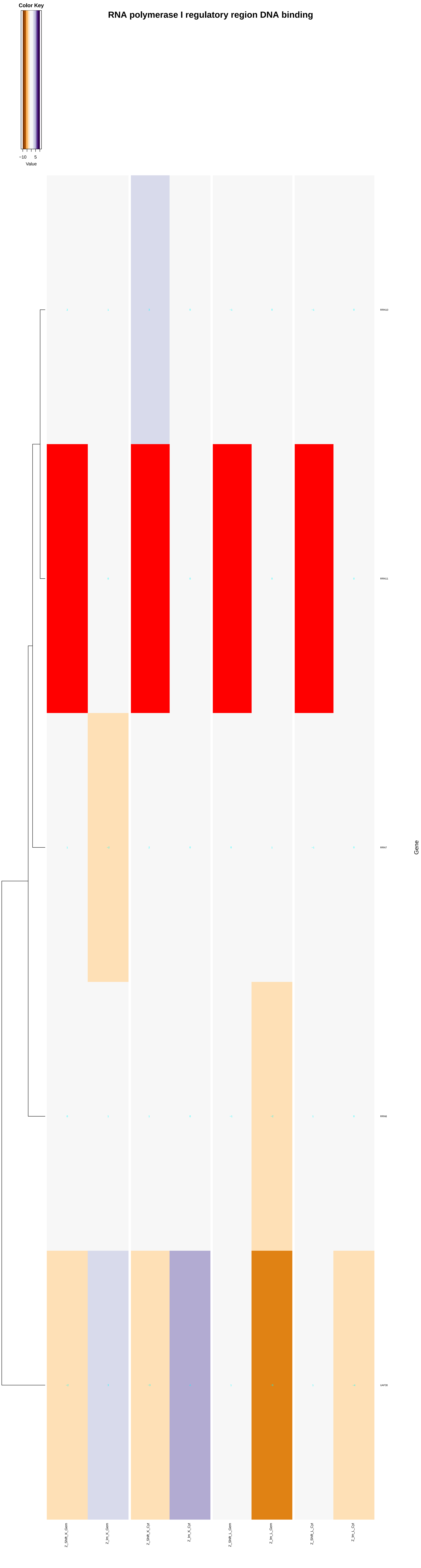

RNA polymerase I regulatory region DNA binding
Color Key
−10
5
Value
RRN10
RRN11
RRN7
RRN6
UAF30
Gene
Z_Shift_K_Gem
Z_Im_K_Gem
Z_Shift_K_Cyt
Z_Im_K_Cyt
Z_Shift_L_Gem
Z_Im_L_Gem
Z_Shift_L_Cyt
Z_Im_L_Cyt

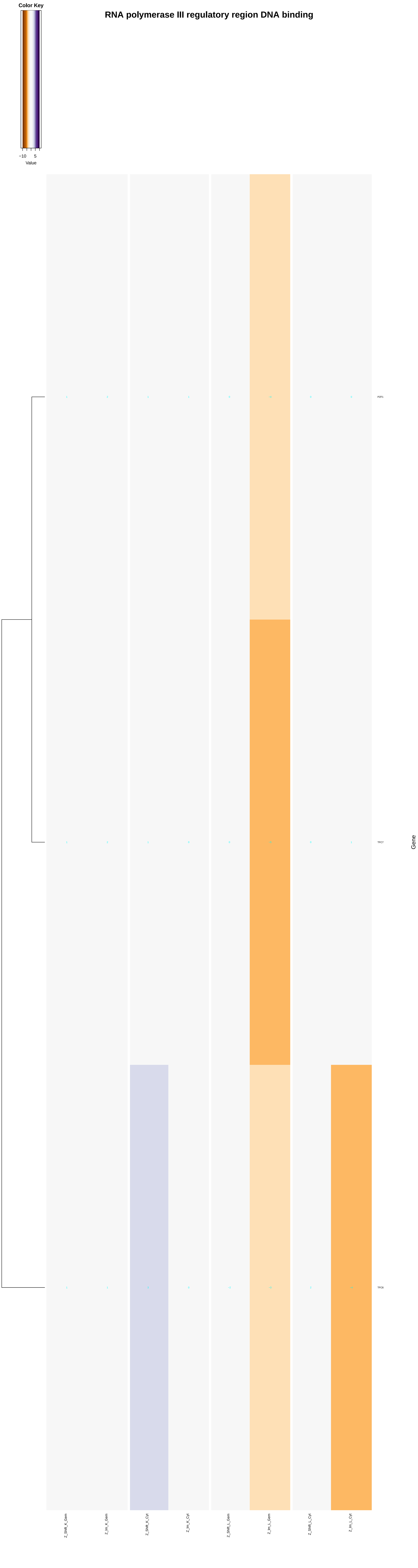

Color Key
−10
5
Value
RNA polymerase III regulatory region DNA binding
PZF1
TFC7
TFC6
Gene
Z_Shift_K_Gem
Z_Im_K_Gem
Z_Shift_K_Cyt
Z_Im_K_Cyt
Z_Shift_L_Gem
Z_Im_L_Gem
Z_Shift_L_Cyt
Z_Im_L_Cyt

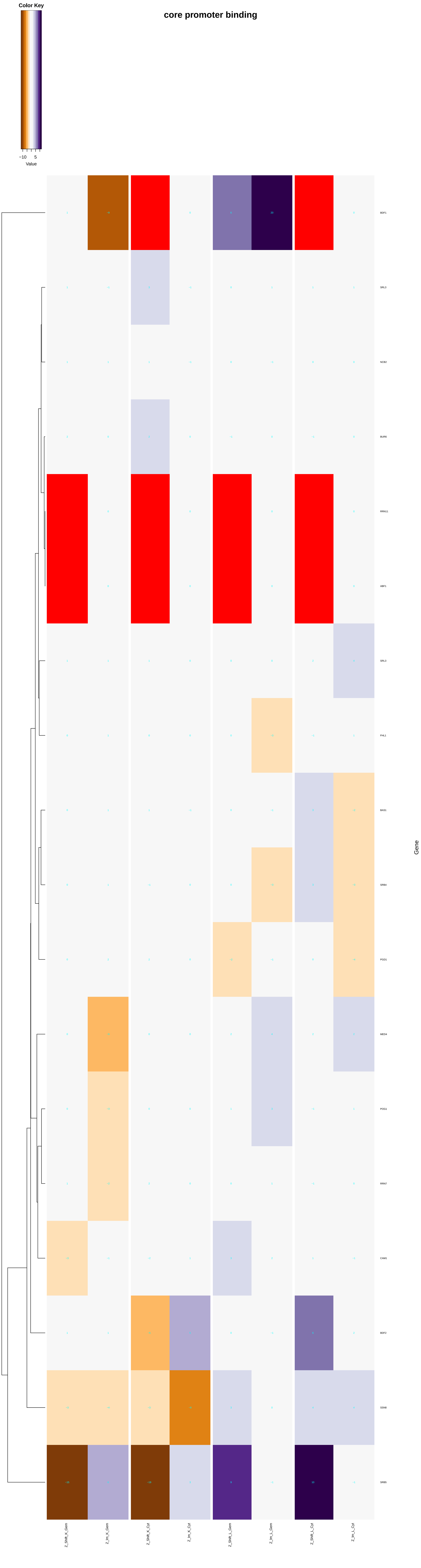
core promoter binding
Color Key
−10
5
Value
BDF1
SRL3
NCB2
BUR6
RRN11
ABF1
SRL3
FHL1
BAS1
SRB4
PGD1
MED4
POG1
RRN7
CAM1
BDF2
SSN8
SRB5
Gene
Z_Shift_K_Gem
Z_Im_K_Gem
Z_Shift_K_Cyt
Z_Im_K_Cyt
Z_Shift_L_Gem
Z_Im_L_Gem
Z_Shift_L_Cyt
Z_Im_L_Cyt

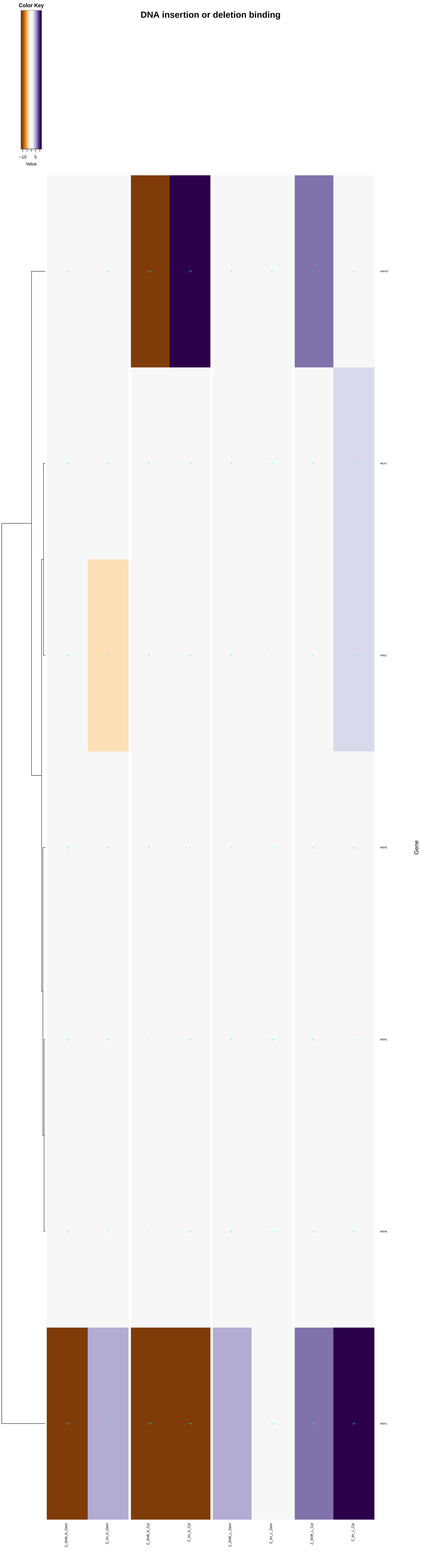
DNA insertion or deletion binding
Color Key
−10
5
Value
NHP10
MLH1
PMS1
MSH3
MSH2
MSH6
MSH1
Gene
Z_Shift_K_Gem
Z_Im_K_Gem
Z_Shift_K_Cyt
Z_Im_K_Cyt
Z_Shift_L_Gem
Z_Im_L_Gem
Z_Shift_L_Cyt
Z_Im_L_Cyt

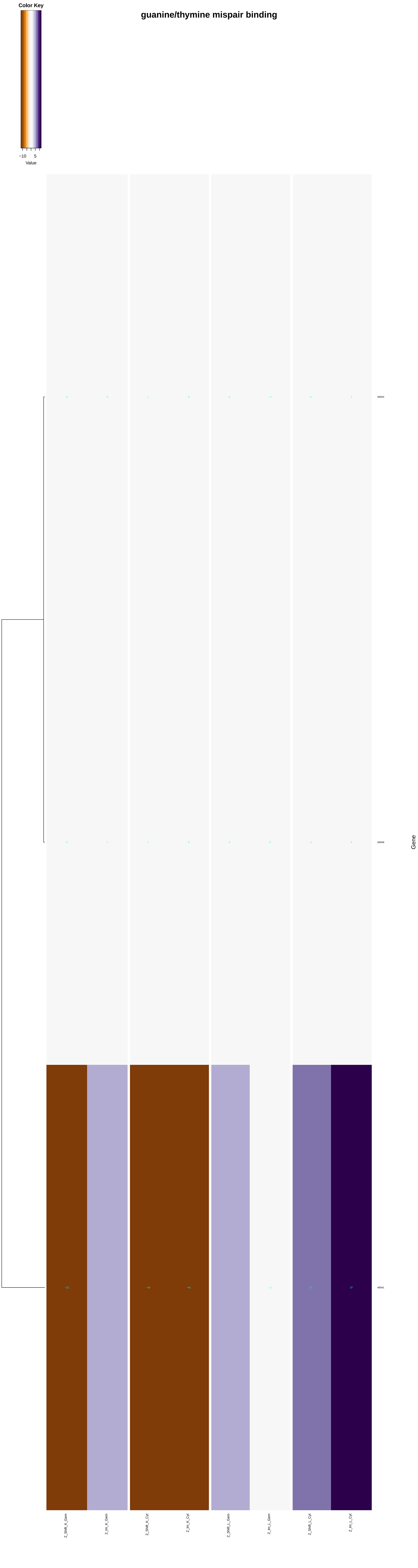
Color Key
−10
5
Value
guanine/thymine mispair binding
MSH2
0 0 1 0 0 −1 0 1
MSH6
0 1 1 0 0 0 −1 0
MSH1
−13 4 −19 −41 6 −1 8 37
Gene
Z_Shift_K_Gem
Z_Im_K_Gem
Z_Shift_K_Cyt
Z_Im_K_Cyt
Z_Shift_L_Gem
Z_Im_L_Gem
Z_Shift_L_Cyt
Z_Im_L_Cyt

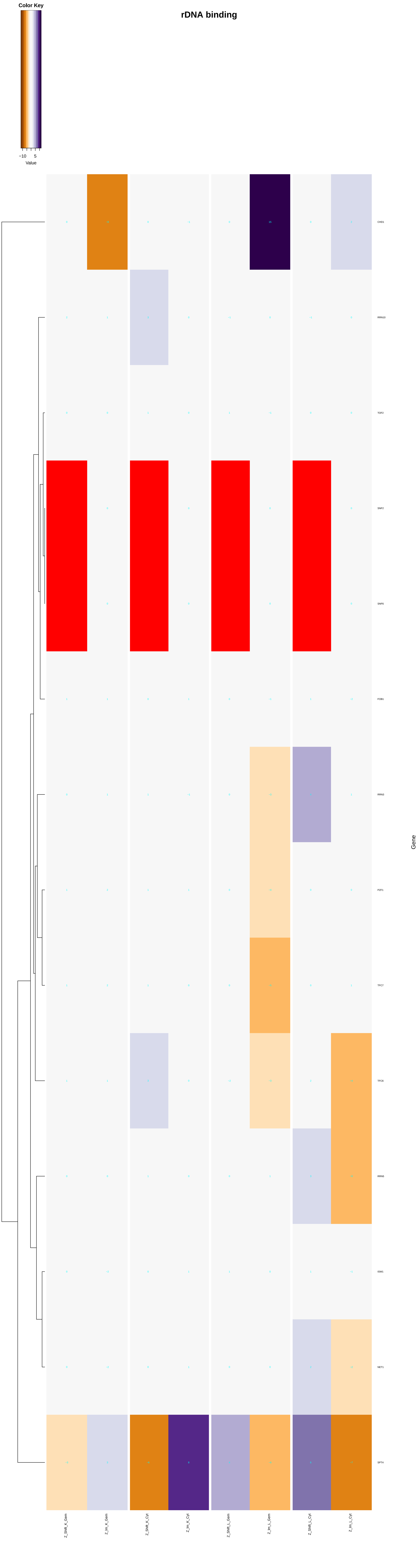

rDNA binding
Color Key
−10
5
Value
CHD1
RRN10
TOF2
SNF2
SNF6
FOB1
RRN3
PZF1
TFC7
TFC6
RRN5
ISW1
NET1
SPT4
Gene
Z_Shift_K_Gem
Z_Im_K_Gem
Z_Shift_K_Cyt
Z_Im_K_Cyt
Z_Shift_L_Gem
Z_Im_L_Gem
Z_Shift_L_Cyt
Z_Im_L_Cyt

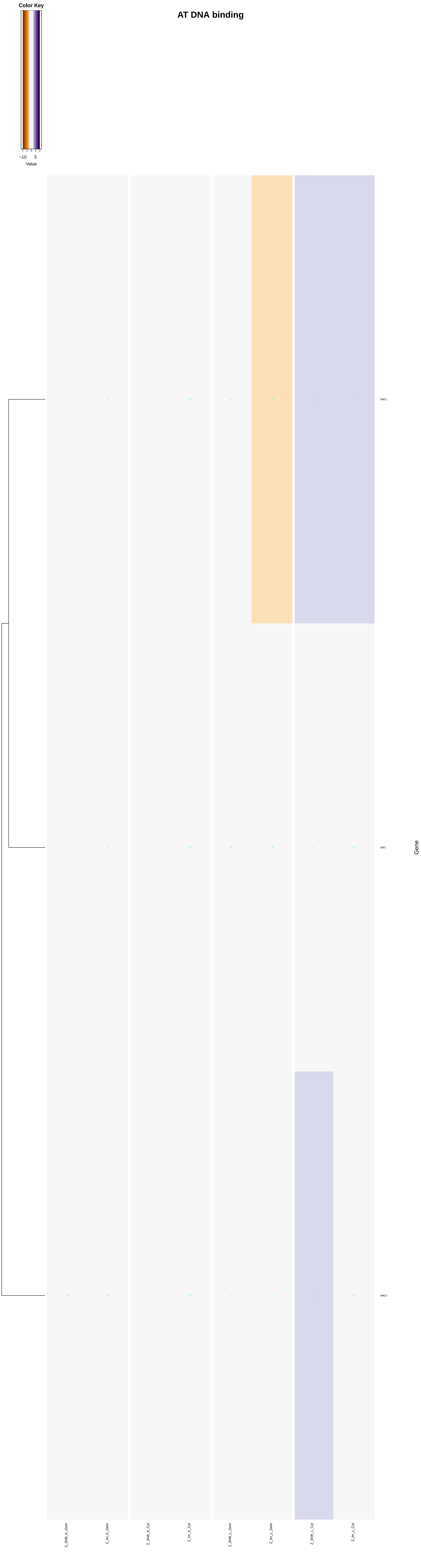
AT DNA binding
Color Key
−10
5
Value
SMC1
DAT1
SMC2
Gene
Z_Shift_K_Gem
Z_Im_K_Gem
Z_Shift_K_Cyt
Z_Im_K_Cyt
Z_Shift_L_Gem
Z_Im_L_Gem
Z_Shift_L_Cyt
Z_Im_L_Cyt

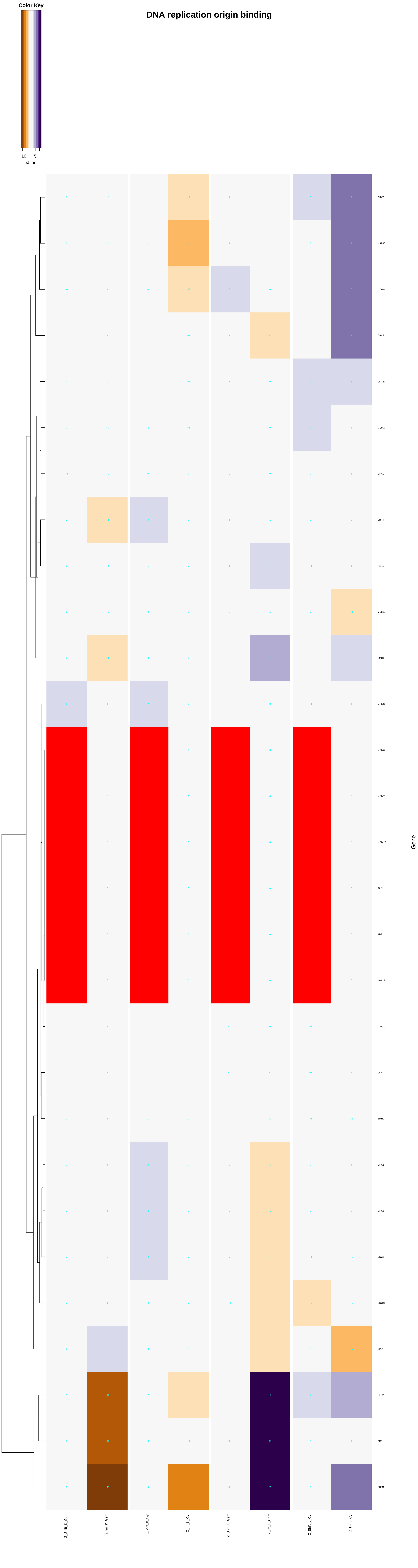

Color Key
−10
5
Value
DNA replication origin binding
ORC6
HSP60
MCM5
ORC3
CDC53
MCM2
ORC2
DBF4
FKH1
MCM1
BMH1
MCM3
MCM6
MCM7
MCM10
SLD2
ABF1
ADE12
TAH11
CLF1
BMH2
ORC1
ORC5
CDC6
CDC45
DIA2
FKH2
BRE1
SUM1
Gene
Z_Shift_K_Gem
Z_Im_K_Gem
Z_Shift_K_Cyt
Z_Im_K_Cyt
Z_Shift_L_Gem
Z_Im_L_Gem
Z_Shift_L_Cyt
Z_Im_L_Cyt

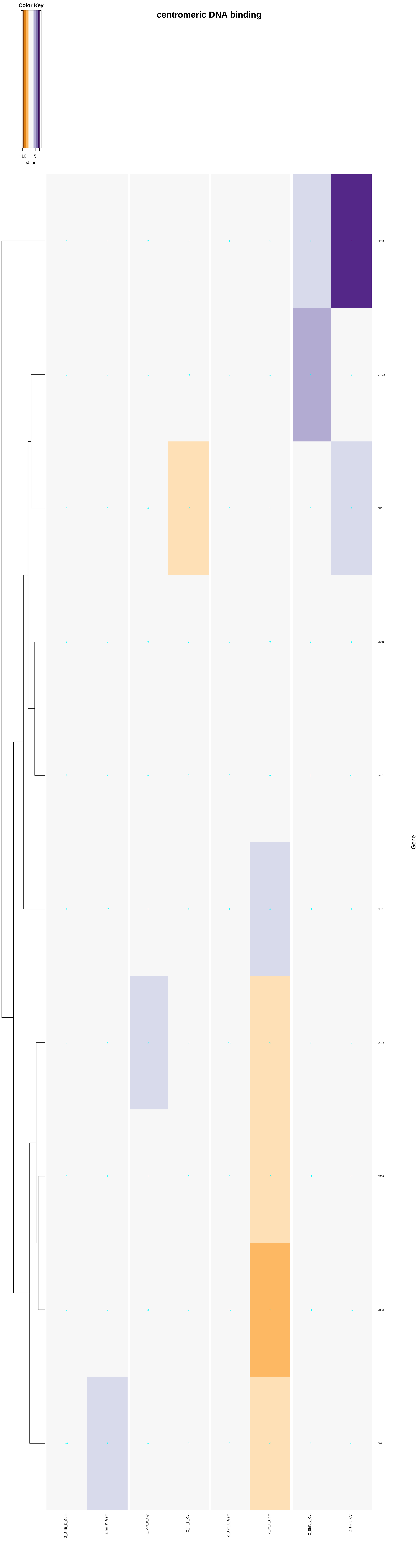

Color Key
−10
5
Value
centromeric DNA binding
CEP3
CTF13
CBF1
CNN1
ISW2
FKH1
CDC5
CSE4
CBF2
CBF1
Gene
Z_Shift_K_Gem
Z_Inv_K_Gem
Z_Shift_K_Cyt
Z_Inv_K_Cyt
Z_Shift_L_Gem
Z_Inv_L_Gem
Z_Shift_L_Cyt
Z_Inv_L_Cyt

single−stranded telomeric DNA binding

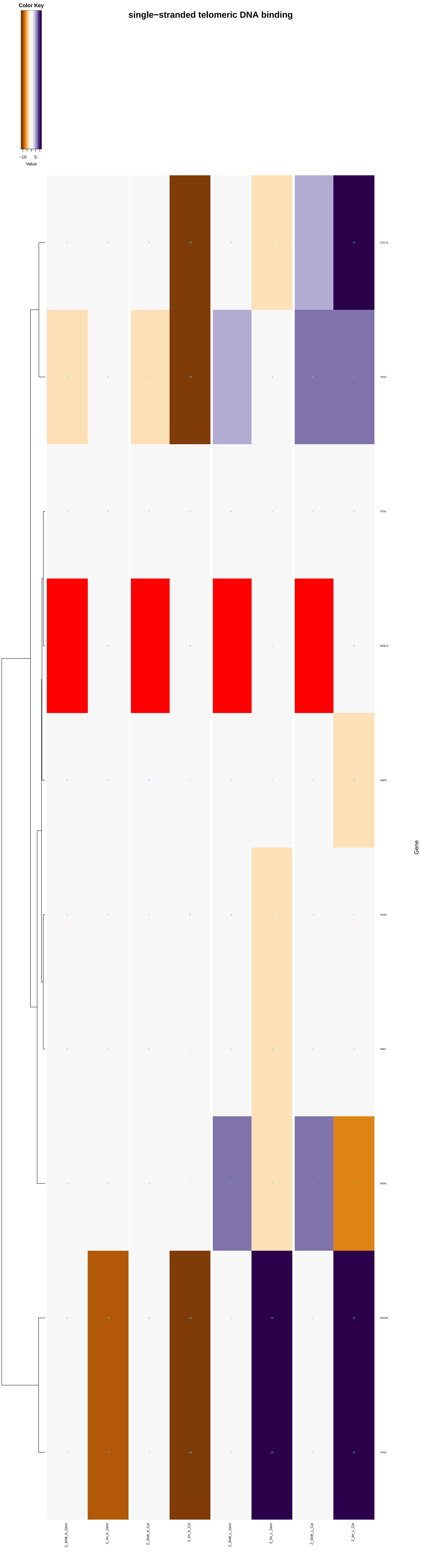

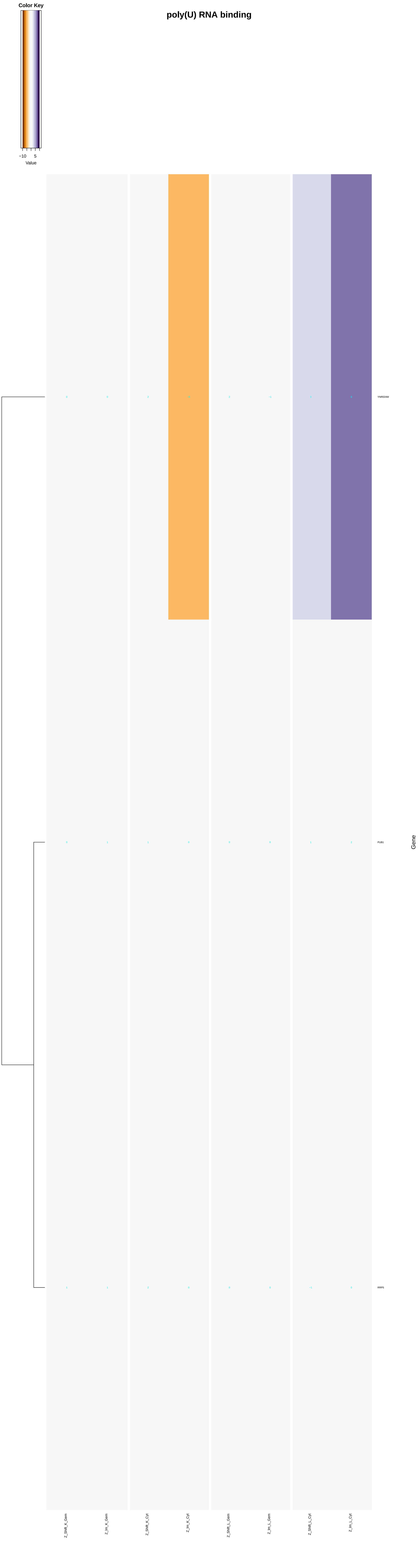
poly(U) RNA binding
Color Key
−10
5
Value
YNR024W
PUB1
RRP5
Gene
Z_Shift_K_Gem
Z_Im_K_Gem
Z_Shift_K_Cyt
Z_Im_K_Cyt
Z_Shift_L_Gem
Z_Im_L_Gem
Z_Shift_L_Cyt
Z_Im_L_Cyt

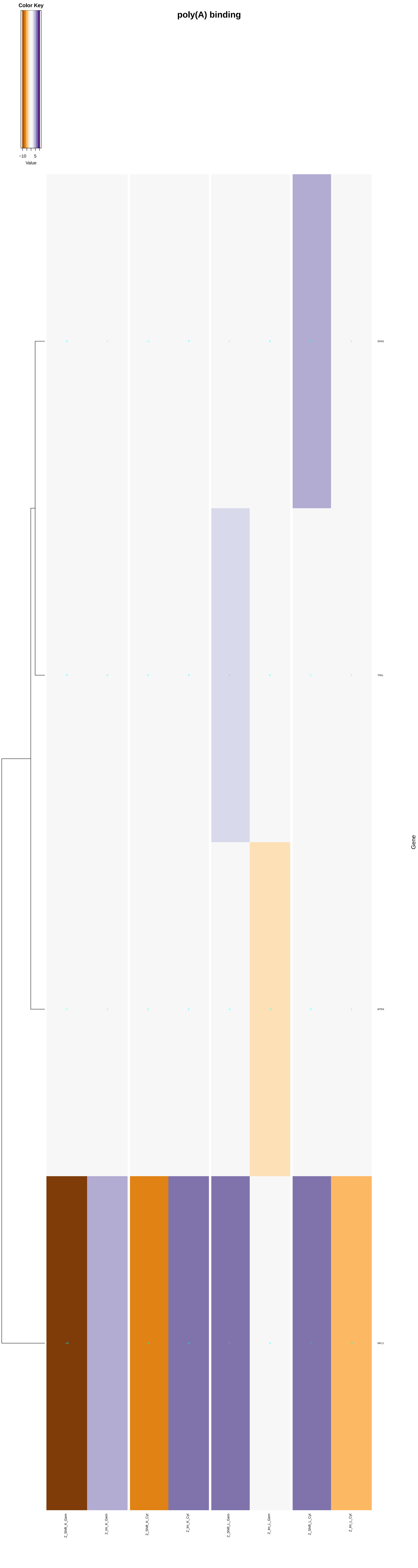

Color Key
−10
5
Value
poly(A) binding
SGN1
TPA1
MTR4
NPL3
Gene
Z_Shift_K_Gem
Z_Int_K_Gem
Z_Shift_K_Cyt
Z_Int_K_Cyt
Z_Shift_L_Gem
Z_Int_L_Gem
Z_Shift_L_Cyt
Z_Int_L_Cyt

translation release factor activity

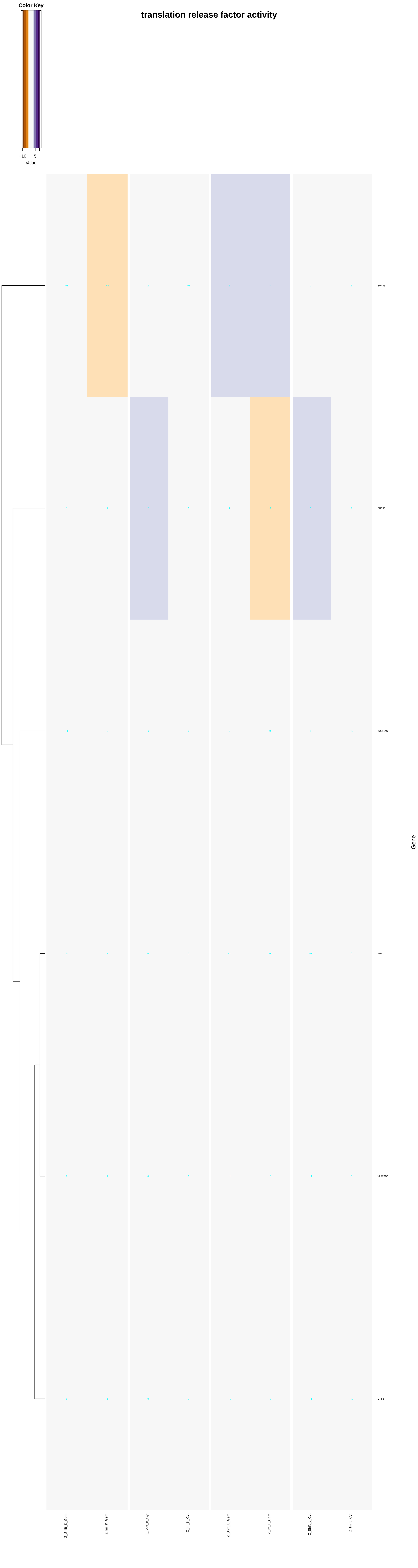

RNA polymerase II regulatory region sequence−specific DNA binding

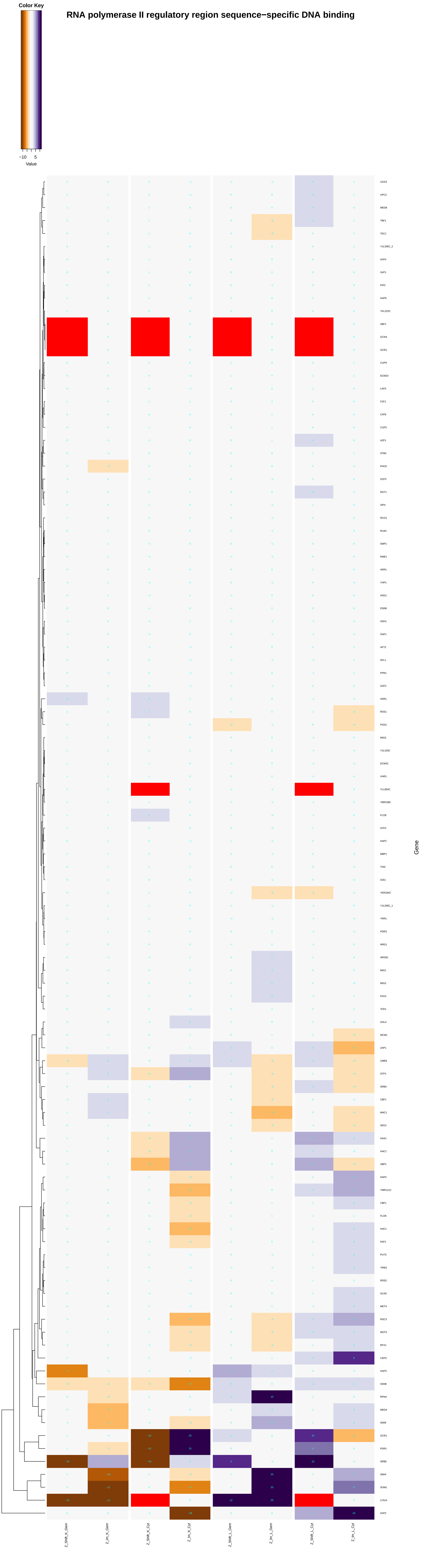

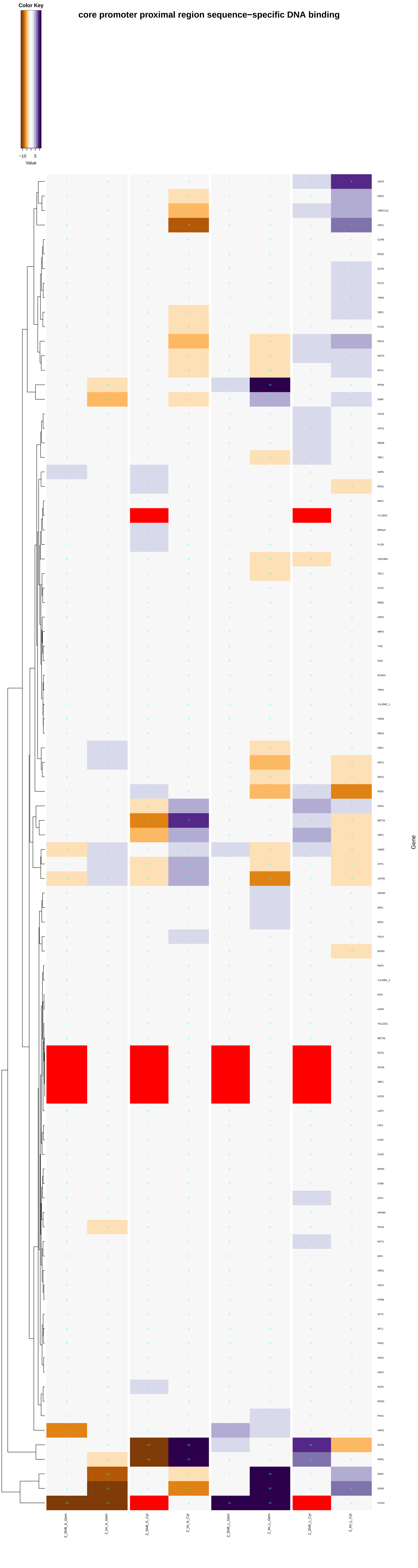
Color Key
−10
5
Value
core promoter proximal region sequence−specific DNA binding
Gene

polymerase III regulatory region sequence−specific DNA binding

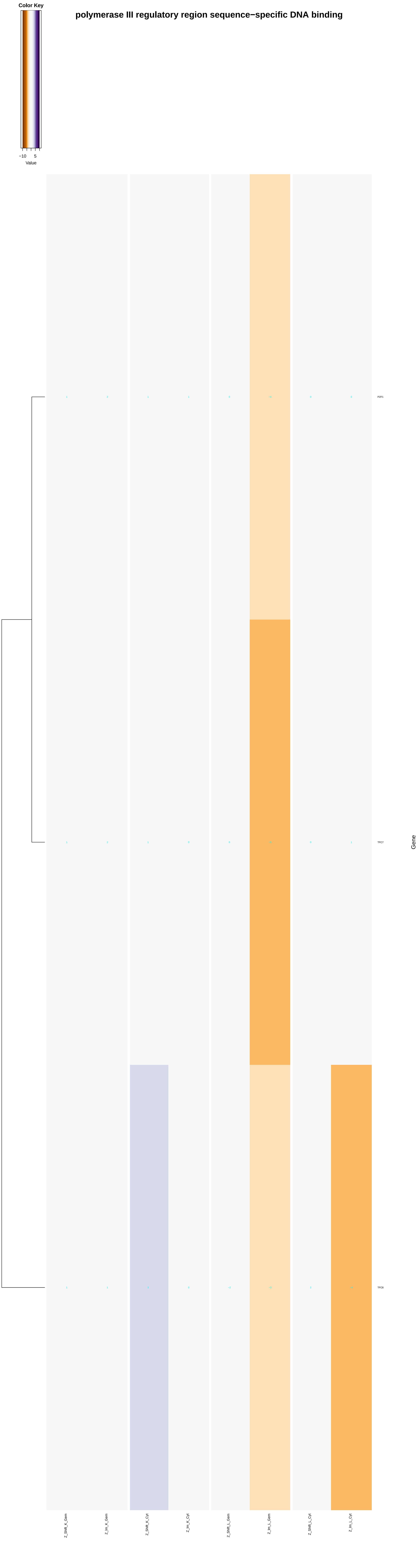

core promoter sequence−specific DNA binding

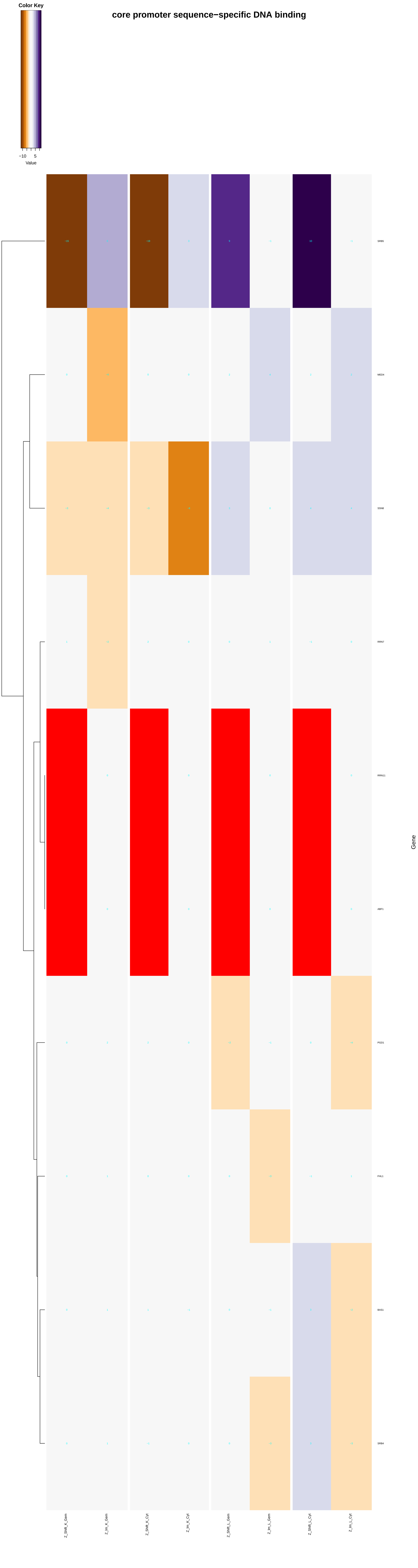

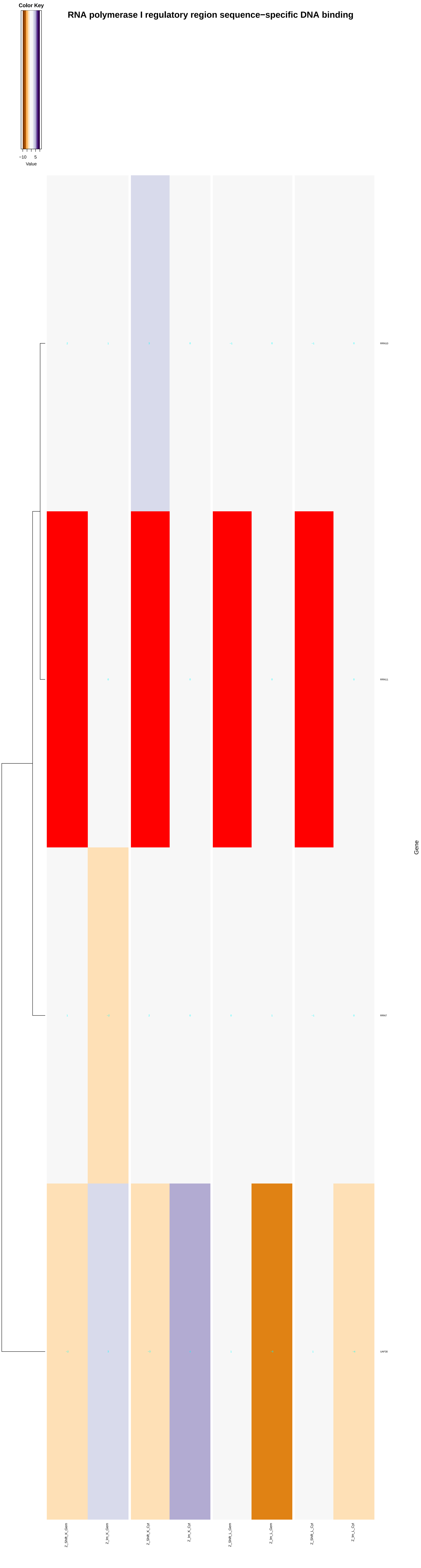
Color Key
−10
5
Value
RNA polymerase I regulatory region sequence−specific DNA binding
RRN10
RRN11
RRN7
UAF30
Gene
Z_Shift_K_Gem
Z_Im_K_Gem
Z_Shift_K_Cyt
Z_Im_K_Cyt
Z_Shift_L_Gem
Z_Im_L_Gem
Z_Shift_L_Cyt
Z_Im_L_Cyt

RNA polymerase III type 1 promoter DNA binding

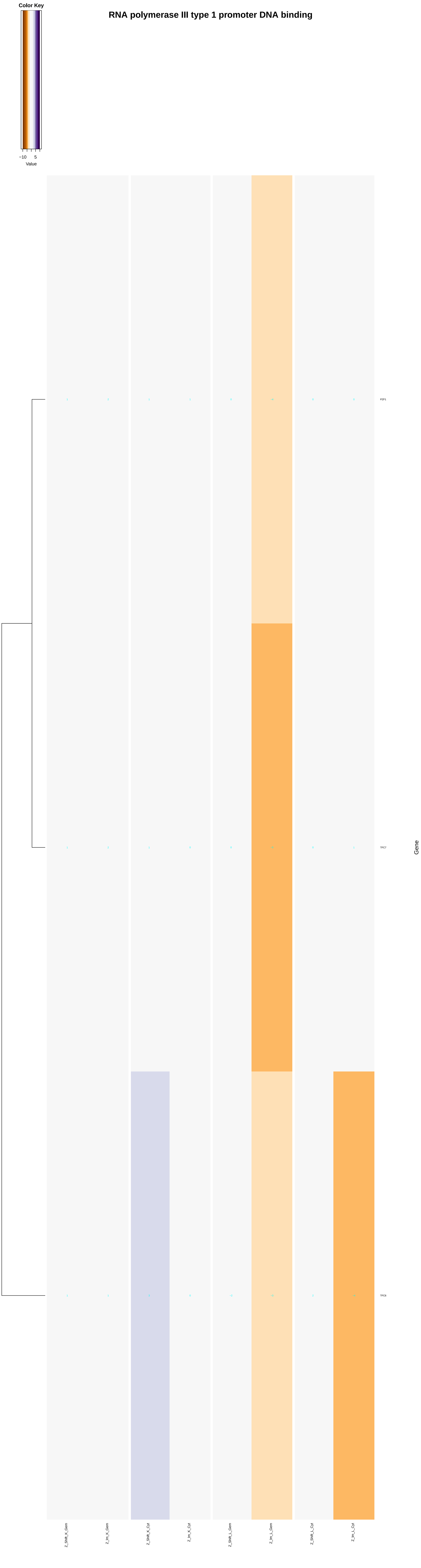

heteroduplex DNA loop binding

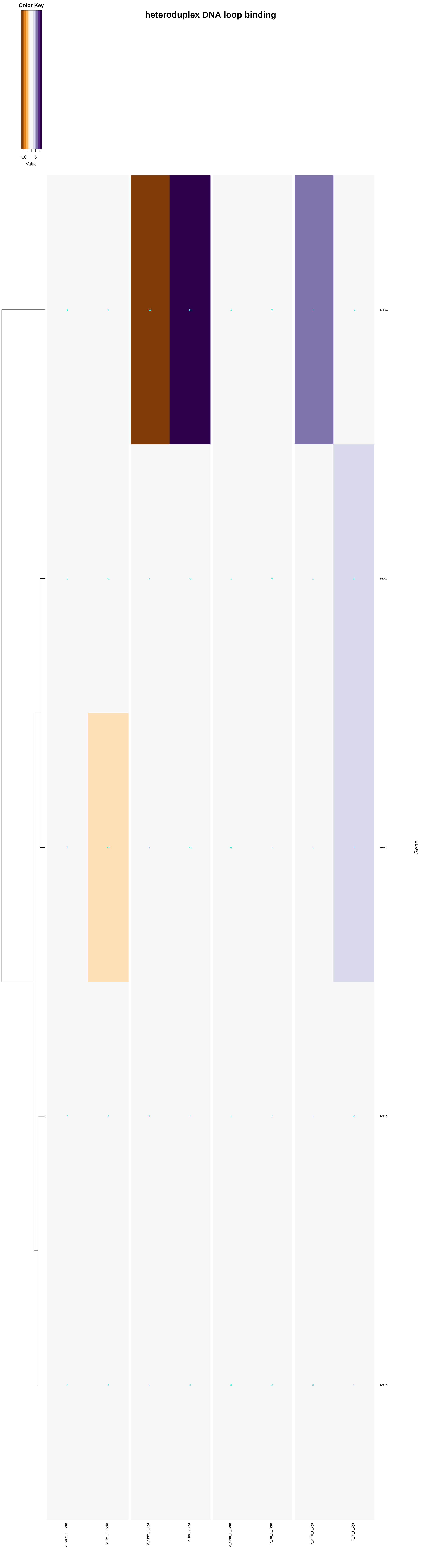

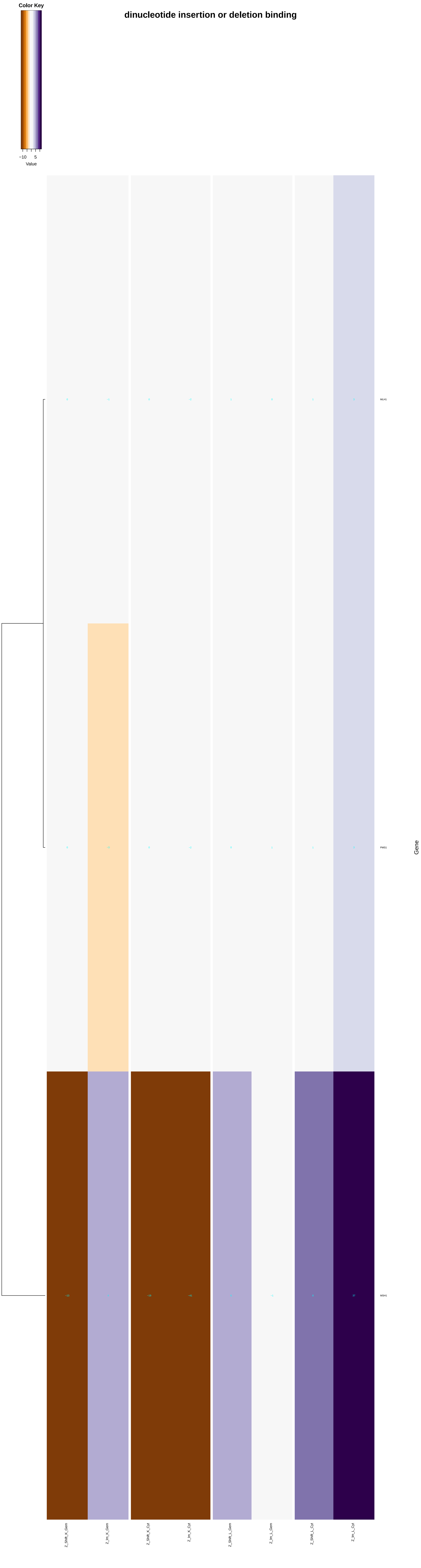

Color Key
−10
5
Value
dinucleotide insertion or deletion binding
MLH1
PMS1
MSH1
Gene
Z_Shift_K_Gem
Z_Im_K_Gem
Z_Shift_K_Cyt
Z_Im_K_Cyt
Z_Shift_L_Gem
Z_Im_L_Gem
Z_Shift_L_Cyt
Z_Im_L_Cyt

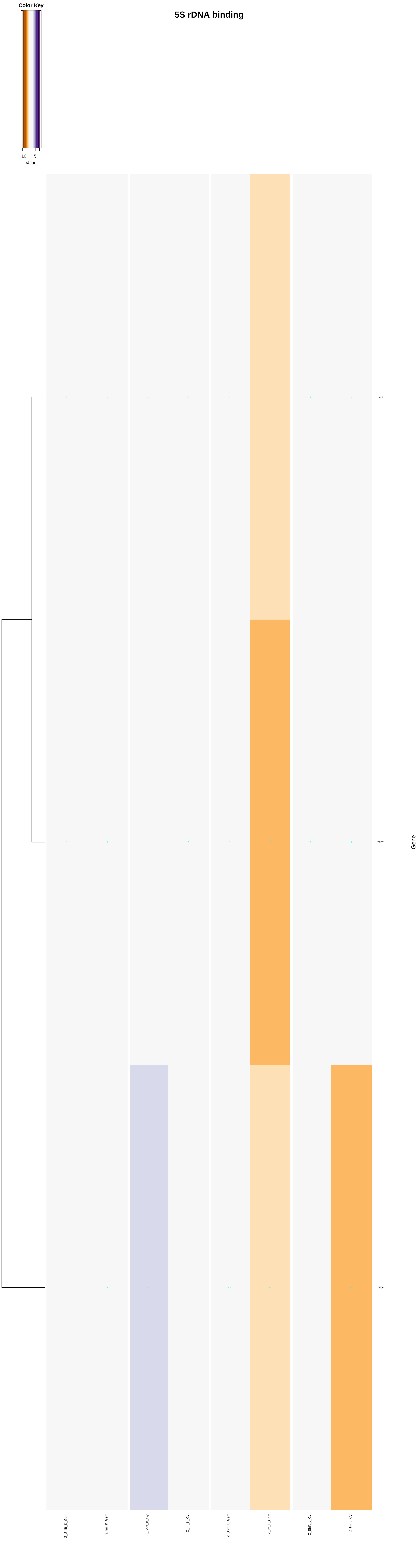

Color Key
−10
5
Value
5S rDNA binding
PZF1
TFC7
TFC6
Gene
Z_Shift_K_Gem
Z_Im_K_Gem
Z_Shift_K_Cyt
Z_Im_K_Cyt
Z_Shift_L_Gem
Z_Im_L_Gem
Z_Shift_L_Cyt
Z_Im_L_Cyt

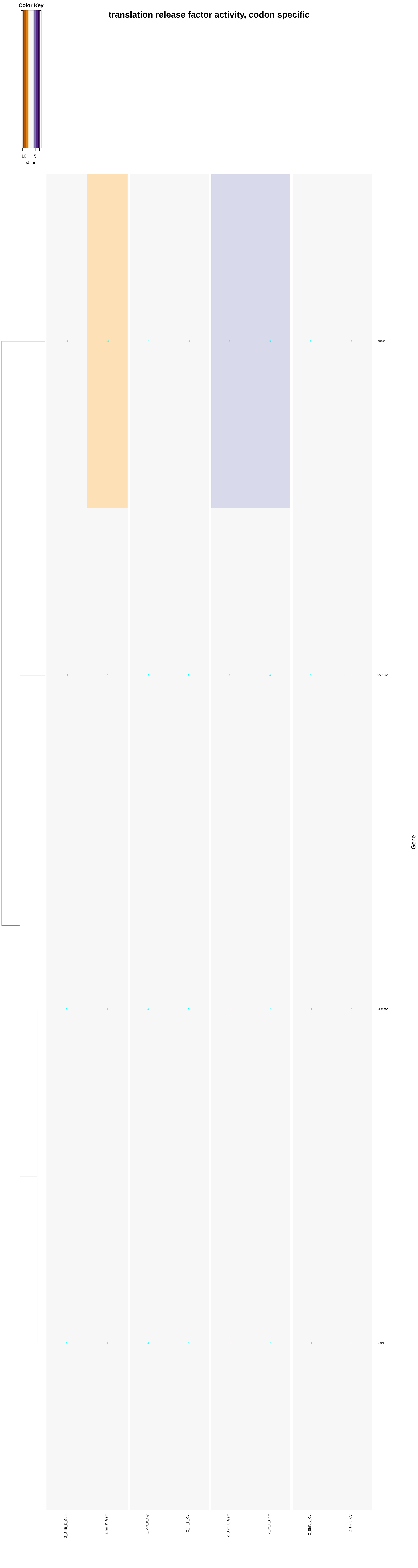

translation release factor activity, codon specific
Color Key
−10
5
Value
SUP45
YOL114C
YLR281C
MRF1
Gene
Z_Shift_K_Gem
Z_lm_K_Gem
Z_Shift_K_Cyt
Z_lm_K_Cyt
Z_Shift_L_Gem
Z_lm_L_Gem
Z_Shift_L_Cyt
Z_lm_L_Cyt

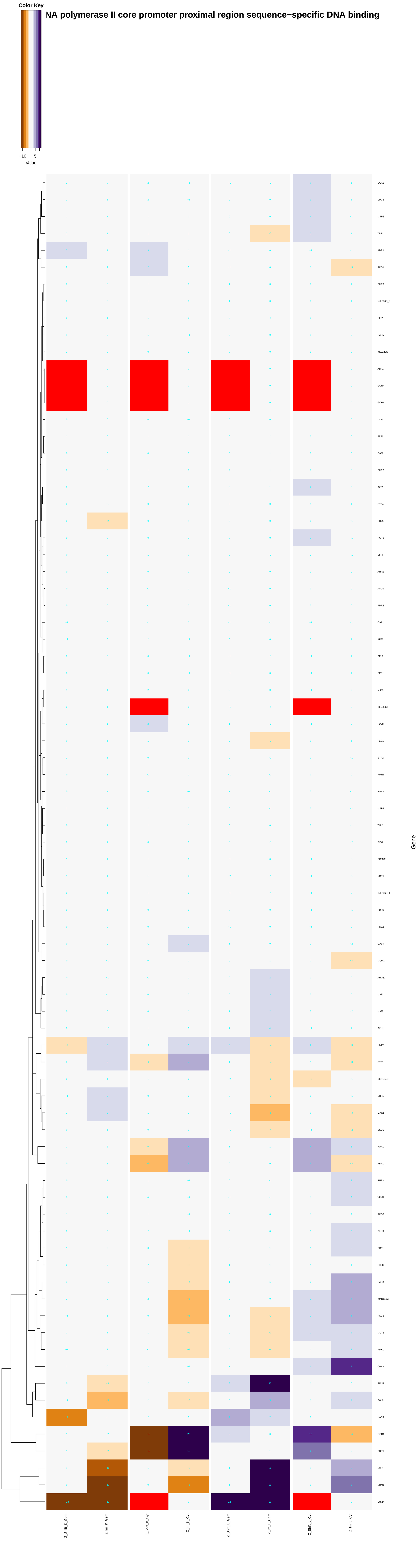
Color Key
−10
5
Value
NA polymerase II core promoter proximal region sequence−specific DNA binding
Gene
Z_Shift_K_Gem
Z_Int_K_Gem
Z_Shift_K_Cyt
Z_Int_K_Cyt
Z_Shift_L_Gem
Z_Int_L_Gem
Z_Shift_L_Cyt
Z_Int_L_Cyt

RNA polymerase II core promoter sequence−specific DNA binding

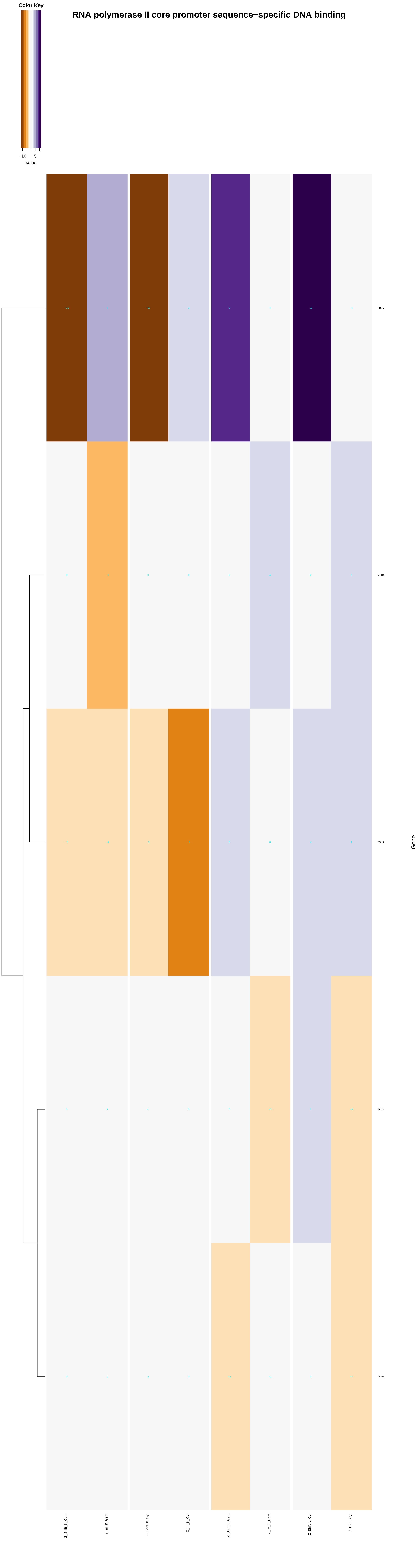

RNA polymerase III type 1 promoter sequence−specific DNA binding

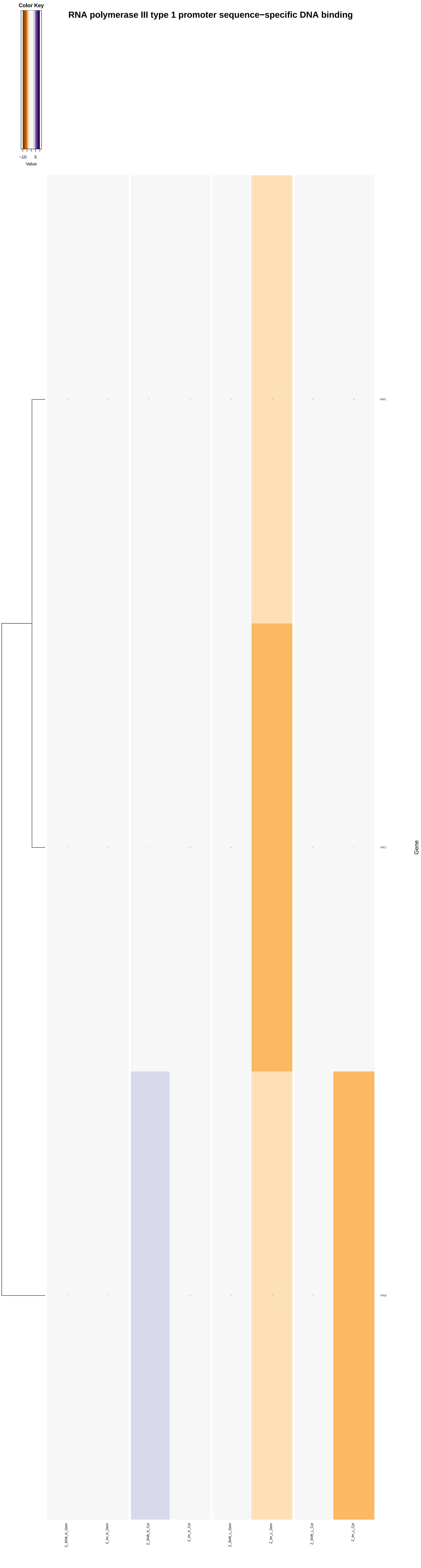

Supplement: Supplementary file 1 [file genes-10-00770-s001.zip › Additional_Files/Additional_File7_GOTermHeatmaps.zip!/Additional_File7_GOTermHeatmaps/nucleic_acid_binding.pdf]
